# Integrating large-scale neuroimaging research datasets: Harmonisation of white matter hyperintensity measurements across Whitehall and UK Biobank datasets

**DOI:** 10.1016/j.neuroimage.2021.118189

**Published:** 2021-08-15

**Authors:** Valentina Bordin, Ilaria Bertani, Irene Mattioli, Vaanathi Sundaresan, Paul McCarthy, Sana Suri, Enikő Zsoldos, Nicola Filippini, Abda Mahmood, Luca Melazzini, Maria Marcella Laganà, Giovanna Zamboni, Archana Singh-Manoux, Mika Kivimäki, Klaus P Ebmeier, Giuseppe Baselli, Mark Jenkinson, Clare E Mackay, Eugene P Duff, Ludovica Griffanti

**Affiliations:** a Wellcome Centre for Integrative Neuroimaging, Oxford Centre for Functional MRI of the Brain, Nuffield Department of Clinical Neurosciences, University of Oxford, Oxford, UK; b Department of Electronics, Information and Bioengineering, Politecnico di Milano, Milan, Italy; c Department of Biomedical, Metabolic and Neural Sciences, University of Modena and Reggio Emilia, Italy; d Wellcome Centre for Integrative Neuroimaging, Oxford Centre for Human Brain Activity, Department of Psychiatry, University of Oxford, Oxford, UK; e Department of Psychiatry, Warneford Hospital, University of Oxford, Oxford, UK; f Department of Biomedical Sciences for Health, Università degli Studi di Milano, Milan, Italy; g IRCCS Fondazione Don Carlo Gnocchi ONLUS, Milan, Italy; h INSERM U1153, Epidemiology of Ageing and Neurodegenerative diseases, Université de Paris, Paris, France; i Department of Epidemiology and Public Health, University College London, London, UK; j Oxford Health NHS Foundation Trust, Oxford, UK; k Department of Paediatrics, University of Oxford, Oxford, UK

**Keywords:** Harmonisation, MRI, White matter hyperintensities, UK Biobank

## Abstract

Large scale neuroimaging datasets present the possibility of providing normative distributions for a wide variety of neuroimaging markers, which would vastly improve the clinical utility of these measures. However, a major challenge is our current poor ability to integrate measures across different large-scale datasets, due to inconsistencies in imaging and non-imaging measures across the different protocols and populations. Here we explore the harmonisation of white matter hyperintensity (WMH) measures across two major studies of healthy elderly populations, the Whitehall II imaging sub-study and the UK Biobank. We identify pre-processing strategies that maximise the consistency across datasets and utilise multivariate regression to characterise study sample differences contributing to differences in WMH variations across studies. We also present a parser to harmonise WMH-relevant non-imaging variables across the two datasets. We show that we can provide highly calibrated WMH measures from these datasets with: (1) the inclusion of a number of specific standardised processing steps; and (2) appropriate modelling of sample differences through the alignment of demographic, cognitive and physiological variables. These results open up a wide range of applications for the study of WMHs and other neuroimaging markers across extensive databases of clinical data.

## Highlights

• We harmonised measures of WMHs across two studies on healthy ageing.
• Specific pre-processing strategies can increase comparability across studies.
• Modelling of biological differences is crucial to provide calibrated measures.

## 1 Introduction

The increasing availability of brain MRI datasets through multi-centre studies, consortia, and data sharing platforms, along with the increased power of computational resources, allows for the possibility of merging datasets and achieving unprecedent statistical power (Smith and Nichols, 2018). This has greatly increased the range of research questions that can now be tackled. Moreover, this provides the possibility of generating normative distributions of neuroimaging markers, which would vastly improve the clinical utility of these measures. However, the increasing use of combined datasets has raised the important issue of ensuring that measures are consistent across datasets. The process of harmonisation aims to remove non-biological variability related to the measurement process, while preserving the biological and especially the clinically-relevant variability present in the data.

In this work we aimed to combine different harmonisation approaches to develop a harmonisation pipeline for MRI-derived measures of white matter hyperintensities (WMHs) of presumed vascular origin (Wardlaw et al., 2013) on two large datasets related to healthy ageing that are part of the Dementias Platform UK (Bauermeister et al., 2020): the Whitehall II imaging sub-study (WH) (Filippini et al., 2014) and the UK Biobank (UKB) (Miller et al., 2016). For the WH study, two MRI scanners were used. A second MRI scanner was included due to a scanner upgrade which took place two-thirds of the way into the study (Zsoldos et al., 2020). The UKB is also focused on the ageing population, but used a different sample demographic, scanner, protocol, and set of non-imaging variables (demographic, cognitive and physiological) compared to WH. Our goal was to find the best combination of processing approaches to minimise non-biological variability in WMH measures extracted from these datasets. This would help providing a comprehensive protocol to successfully reduce biases and promote data integration.

The importance of characterising ageing-associated vascular damage is increasingly recognised, since vascular disease contributes to more than half of dementia cases, often in conjunction with Alzheimer's disease pathology (Arvanitakis et al., 2016; Debette et al., 2010). Among the signs of cerebral small vessel disease (SVD), WMHs are one of the most commonly evaluated, but their underlying pathology and clinical impact on cognition is still poorly understood (Wardlaw et al., 2013), and possibly affected by age (Zamboni et al., 2019). An ability to combine datasets would give additional insight on the relationships between WMHs, its risk factors and clinical outcomes. It would not only improve statistical power, but also enable complementary information from datasets to be merged. For example, WH includes detailed longitudinal cognitive and behavioural assessments that are of great importance for understanding dementia pathology. On the other hand, the UKB dataset has a larger sample size, wider age range and more even gender balance than WH, providing data on a wider segment of the population. An ability to integrate WMH data across these two datasets would combine their strengths and lead to novel insights into the prognostic value of WMHs.

While many harmonisation approaches have been developed and tested on T1-weighted (e.g. Fortin et al., 2018; Zandifar et al., 2018) and diffusion MRI (e.g. Fortin et al., 2017; Mirzaalian et al., 2016), studies evaluating harmonisation approaches for T2-weighted scans and the quantification of WMHs (and other lesions) are still lacking, despite the recognition that biases are also present in this modality (Shinohara et al., 2017; Guo et al., 2019). Consortia and working groups (Wardlaw et al., 2013; Smith et al., 2019) recognised the need to standardise the assessment of cerebral SVD and proposed a set of standard definitions, acquisition protocols and a framework for developing neuroimaging biomarkers of the condition. The HARmoNising Brain Imaging MEthodS for VaScular Contributions to Neurodegeneration (HARNESS) initiative (https://harness-neuroimaging.org) also provides web-based repositories of protocols, software tools and rating scales to facilitate multi-centre research. While all these resources contribute to more standardised assessment of WMHs, what is still currently lacking is a way to make quantitative measures truly consistent.

The datasets we selected for this study allow us to test retrospective (i.e. after data collection) harmonisation strategies in the presence (WH scanner upgrade) and absence (WH-UKB) of prospective (i.e. prior to data collection) harmonisation. Harmonised acquisition protocols are commonly employed in consortia and multi-centre studies (Jack et al., 2008; Potvin et al., 2019) to facilitate future integration or comparison of data, with agreement on collection procedures and common measures prior to data collection. However, even after careful protocol harmonisation, systematic differences in images across sites can remain (related to scanner vendor, model, non-linearity of imaging gradients, magnetic field homogeneity, signal-to-noise ratio etc.) and lead to bias in the MRI-derived measures (Kruggel et al., 2010; Potvin et al., 2019; Shinohara et al., 2017; Mirzaalian et al., 2016; Guo et al., 2019). At the image pre-processing level, harmonisation strategies aim to directly remove the non-biological variability in the images (Mirzaalian et al., 2016; Dewey et al., 2018) and provide processing procedures that ensure well-matched measures and consistent performance across datasets (Erus et al., 2018; Zandifar et al., 2018; Guo et al., 2019). At the analysis level, harmonisation approaches may further standardise measures derived across datasets and account for differences in the samples across studies, to ensure that characteristics of imaging site and study do not bias analyses (Fortin et al., 2017; Fortin et al., 2018; Pomponio et al., 2020). Despite rapid progress, MRI data harmonisation remains a challenge because of the many sources that may drive variability in MR measurements across datasets. Due to the different nature of the biases involved, a single strategy is unlikely to achieve successfully harmonised data (Wachinger et al., 2019; Glocker et al., 2019). With this study we aim to combine manifold approaches to overcome such limitation.

A key element of the present work involved increasing the robustness of FSL-BIANCA, a supervised classification method for segmenting WMHs (Griffanti et al., 2016). Briefly, BIANCA classifies the image voxels based on their intensity and spatial features using the k-nearest neighbour (k-NN) algorithm. The intensity features used by BIANCA can be extracted from multiple MRI modalities, making it a very versatile tool. Being a supervised method, it needs examples of manually segmented WMHs for training the algorithm. The output image represents the probability of each voxel being a WMH. This image can then be thresholded to obtain the final binary mask representing the WMHs (see Griffanti et al., 2016 for further details). BIANCA has been tested on vascular, neurodegenerative and healthy populations. It achieved excellent performance scores with respect to manual annotation and visual rating. It is registered among the software tools on the HARNESS initiative website (https://software.harness-neuroimaging.org/harness-software-catalog/bianca.html).

We assessed the effects of a variety processing choices on the harmonisation of the identification of WMHs. As different datasets will often have different imaging modalities available, we determined the effects of using different combinations of modalities to train BIANCA. As the rater employed to perform the manual segmentations used to train BIANCA will typically be different across studies, we assessed impact of the choice of rater, and the effects of training BIANCA on individual datasets or on combined data. We also assessed the effect of using bias field correction, as the distribution of radio frequency field (RF) inhomogeneities is unique to each scanner. We first explored the impact of these processing choices on the harmonisation of WMHs measured across a scanner change, using the WH data. We then extended the evaluation to the retrospective merging of data across studies, comparing WH (as a whole) with the UKB. Particularly in the latter case, it was important to include non-imaging variables such as age and sex, to account for differences in study populations. Finally, based on the results of these assessments, we propose a set of recommendations for improving WMH comparability across datasets.

## 2 Methods

### 2.1 Datasets

The datasets we used in this work are WH and UKB.

The first, described in Filippini et al., 2014, is part of a large longitudinal study, namely the Whitehall II Study, that explores the social determinants of health. It involves a sample of British civil servants (age range 60–85 years) who were first recruited in 1985 and participated in a number of phases of clinical/cognitive assessment. Seven hundred and seventy-four participants were selected randomly to receive multi-modal brain MRI scans and a detailed cognitive battery at the Oxford Centre for Functional MRI of the Brain (FMRIB) as part of the Imaging Sub-study (2012–2016). Out of those, we excluded 18 participants with evident brain abnormalities other than WMHs (e.g. tumour, stroke, multiple sclerosis), 17 due to poor quality of the available MRI scans or lack of some of the MRI contrast of interest, and 26 with missing data in the non-imaging variables of interest (details below). As a result, the analysis was performed on a total number of 713 subjects, of which 513 (WH1) were imaged with a 3T Siemens Verio scanner (SC1) and 200 (WH2) with a 3T Siemens Prisma (SC2). Alongside the WH cohort, 5 additional young and healthy participants (age 31 ± 4.9 years, age range 26–39 years, 2 males) were also scanned at FMRIB on both SC1 and SC2 (‘traveling heads’). They were all acquired immediately before the scanner replacement and then as soon as possible after the installation of the new machine, with an average time between scans of 5 months (150 ± 6 days). The time of the day was not necessarily the same for all subjects (details in Supplementary Table S1). Even though these subjects did not have any WMHs, the MRI data allowed us to get additional insight on non-biological sources of variability in the images and test some harmonisation approaches.

The second dataset is the UKB imaging study, a sub-study of the UKB prospective epidemiological study gathering extensive questionnaires, physical and cognitive measures, and biological samples from predominantly healthy participants. The project imaging component Miller et al., 2016, currently ongoing, aims to collect detailed diagnostic MRI scans from 100,000 UKB participants. The sample available at the time of our work included 14,503 subjects with scans released by January 2019 (age range 46–80 years). Out of those with available MRI data, we selected 2,295 participants who had no missing data in the non-imaging variables of interest (details below). This allowed us to avoid performing data imputation, which could have introduced an additional source of variability, while retaining a large number to focus on the methodological goal of imaging data harmonisation. Ten further participants were excluded due to other brain abnormalities. The resulting UKB dataset was therefore composed of 2,285 participants.

*Non-imaging variables* – In order to model the biological variability in WMH measures across datasets, we selected non-imaging variables with a potential link to WMHs. An example of such variables is age. It is one of the most important risk factor for WMHs, and WH and UKB have only partially overlapping age ranges (WH: 60–85 years; UKB: 46–80 years). Therefore, we considered particularly important to take it into account as source of biological variability when comparing measures of WMHs across datasets. A total of 33 variables, including demographic, clinical and cognitive factors were selected among those available for the WH dataset. Subsequently, when performing harmonisation between the WH and UKB datasets, we excluded 4 variables due to lack of availability for all participants within the UKB cohort, or due to substantial differences in the data collection across the two datasets (e.g. the design or administration of certain cognitive tests). The full list of non-imaging variables selected for both datasets is presented in Table 1.

**Table 1:** Details of the non-imaging variables selected for our study.

	Variables	Raw Units		Harmonised Units chosen	Value harmonised		
		Whitehall	UK Biobank		Whitehall	UK Biobank	
Demographic	**Age**	Years (continuous)	Years (integer)	Years (integer)	69.73 ± 5.15	61.46 ± 7.13	**
	Sex	Categorical (binary)	Categorical (binary)	Categorical (binary)	M: 586 (82.19%)	M: 1013 (44.33%)	**
Biological	Weight	kg (continuous)	kg (continuous)	kg (continuous)	78.72 ± 13.69	74.81 ± 14.68	**
	**Height**	m (continuous)	cm (integer)	m (continuous)	1.74 ± 0.08	1.70 ± 0.09	**
	BMI (Body Mass Index)	Kg/m^2^ (continuous)	Kg/m^2^ (continuous)	Kg/m^2^ (continuous)	26.11 ± 4.09	26.05 ± 4.31	
	Systolic blood pressure	mmHg (integer)	mmHg (integer)	mmHg (integer)	141.31 ± 17.44	136.94 ± 19.03	**
	Diastolic blood Pressure	mmHg (integer)	mmHg (integer)	mmHg (integer)	77.51 ± 10.71	78.10 ± 10.44	
	Pulse	bpm (integer)	bpm (integer)	bpm (integer)	67.57 ± 12.03	70.73 ± 12.09	**
	**Hand class**	Categorical (3 classes)	Categorical (4 classes)	Categorical (3 classes)	Right: 631 (88.50%), Left: 59 (8.27%), Ambidextrous: 23 (3.23%)	Right: 2034 (89.01%), Left: 211 (9.24%), Ambidextrous: 40 (1.75%)	
Socioeconomic	Education	Years (int)	N/A	N/A	19.10 ± 2.85	N/A	
Health behaviours	**Moderate physical activity**	h/week (continuous)	day/week (integer), min/day (integer)	h/week (continuous)	16.99 ± 27.59	4.27 ± 5.96	**
	**Vigorous physical activity**	h/week (continuous)	min/day (integer)	h/week (continuous)	9.41 ± 17.06	1.47 ± 2.27	**
	**Combination of different motorial tasks**	h/week (continuous)	day/week (integer), min/day (integer)	h/week (continuous)	25.16 ± 34.87	5.32 ± 6.79	**
	**Time spent watching TV**	h/week (continuous)	h/day (integer)	h/week (integer)	5.62 ± 3.28	19.07 ± 10.09	**
	**Total walking activity**	h/week (continuous)	min/day (integer)	h/week (continuous)	10.10 ± 8.00	6.36 ± 6.68	**
	**Sleep duration**	h/day (continuous)	h/day (integer)	h/day (integer)	6.92 ± 1.01	7.21 ± 0.96	**
	**Smoker status**	Categorical (binary)	Categorical (4 classes)	Categorical (binary)	Smoker: 27 (3.79%)	Smoker: 65 (2.84%)	
	Cigarette units	units/day (integer)	units/day (integer)	units/day (integer)	0.45 ± 2.84	0.32 ± 2.20	
	**Alcohol status**	Categorical (binary)	Categorical (4 classes)	Categorical (binary)	Consumer: 639 (89.62%)	Consumer: 2206 (96.54%)	**
	**Alcohol units**	units/month (continuous)	units/day (categorical, 5 classes), day/week (categorical, 5 classes)	units/month (continuous)	14.83 ± 15.16	5.26 ± 39.05	**
CVD (cardiovascular disease)	**Medications for Cardiovascular Disease**	Categorical (binary)	Categorical (6 classes)	Categorical (binary)	Yes: 381 (53.44%)	Yes: 228 (9.98%)	**
	**History of Cardiovascular Disease**	Categorical (binary)	Categorical (6 classes)	Categorical (binary)	Yes: 133 (18.65%)	Yes: 442 (19.34%)	
General health	**Self-rated health**	Categorical (4 classes)	Categorical (9 classes)	Categorical (4 classes)	Poor: 6 (0.84%), Fair: 52 (7.29%), Good: 224 (31.42%), Very good/Excellent: 431 (60.45%)	Poor: 17 (0.74%), Fair: 249 (10.90%), Good: 1425 (62.36%), Very good/Excellent: 594 (26.00%)	**
	Total number of medications	units (integer)	units (integer)	units (integer)	2.84 ± 2.49	1.60 ± 1.81	**
	**Medications for Blood Pressure**	Categorical (binary)	Categorical (6 classes)	Categorical (binary)	Yes: 232 (32.54%)	Yes: 227 (9.93%)	**
	**History of Diabetes**	Categorical (binary)	Categorical (4 classes)	Categorical (binary)	Yes: 62 (8.70%)	Yes: 68 (2.98%)	**
Mental health	**Center for Epidemiologic Studies-Depression (CES-D) scale**	Categorical (4 classes)	Categorical (5 classes)	Categorical (4 classes)	Not at all: 600 (84.15%), Several days: 90 (12.62%), More than half the days: 18 (2.53%), Nearly every day: 5 (0.70%)	Not at all: 1879 (82.23%), Several days: 362 (15.84%), More than half the days: 28 (1.23%), Nearly every day: 16 (0.70%)	*
	Depression - Medications	Categorical (binary)	N/A	N/A	Yes: 29 (4.07%)	N/A	
Cognitive skills	**Trail Making Test (TMT) A**	seconds (integer)	seconds (continuous)	seconds (integer)	30.69 ± 11.10	37.72 ± 13.46	**
	**Trail Making Test (TMT) B**	seconds (integer)	seconds (continuous)	seconds (integer)	66.75 ± 33.72	62.15 ± 22.16	**
	Digit CODing (DCOD)	Correct answers (integer)	~	~	63.13 ± 13.01	~	
	Digit Span Backward (DSB)	*u* (integer)	*u* (integer)	*u* (integer)	9.67 ± 2.44	7.07 ± 1.42	**
	Reaction time	ms (continuous)	~	~	315.45 ± 68.03	~	

For each variable we display the raw units (used at the time of data collection), the units chosen to harmonise the data, and the numerical values for the two cohorts in harmonised units, which allowed us to compare the two cohorts. The last column displays the results of the tests (*t*-test or chi-square, as appropriate) showing non-imaging differences between the two cohorts (* for *p*-values < 0.05 and ** for *p*-values < 0.01). Variables requiring the application of non-imaging harmonisation strategies are highlighted in bold. Legend: N/A = excluded due to lack of availability for all participants within the UK Biobank cohort, ~ = excluded due to substantial differences in the data collection across the two datasets.

*MRI data acquisition* – Acquisition details for the datasets involved in our analysis are listed in Table 2.

**Table 2:** Acquisition details for the three scanners involved in our study.

	Whitehall						UK Biobank	
	3T Siemens Verio (WH1)			3T Siemens Prisma (WH2)			3T Siemens Skyra (UKB)	
Sequence	FLAIR	T1 (MEMPR)	dMRI (EPI)	FLAIR	T1 (MPRAGE)	dMRI (EPI)	FLAIR	T1
TR (ms)	9000	2530	8900	9000	1900	8900	5000	2000
TE (ms)	73	1.79/3.65/5.51/7.37	91.2	73	3.97	91	395.0	2.01
Flip angle (degrees)	150	7	—–	150	8	—–	—–	8
Voxel dimension (mm^3^)	0.9x0.9x3	1x1x1	2x2x2	0.4x0.4x3	1x1x1	2x2x2	1.05x1x1	1x1x1
FoV read (mm)	220	256	192	220	192	192	256	256
FoV phase (%)	100	100	100	100	100	100	100	100
Base resolution	256	256	96	256	256	96	256	256
Phase resolution (%)	100	100	100	100	100	100	100	100
TI (ms)	2500	1380	—–	2500	904	—–	1800	880
Bandwidth (Hz/Px)	283	651	1680	283	200	1680	781	240
Orientation	Transversal	Sagittal	Transversal	Transversal	Transversal	Transversal	Sagittal	Sagittal
b-value (s/mm^2^)	—–	—–	1500	—–	—–	1500	—–	—–
Directions (n.)	—–	—–	60 + 6 b0 (1 reversed PE)	—–	—–	60 + 6 b0 (1 reversed PE)	—–	—–
Acquisition time	4m 14s	6m 12s	9m 56s	4m 14s	5m 31s	10m 41s	5m 52s	4m 54s

Legend: FLAIR, fluid attenuated inversion recovery; MEMPR, Multi-Echo MPRAGE; MPRAGE, Magnetisation Prepared Rapid Acquisition Gradient Echo; dMRI, diffusion MRI; EPI, Echo Planar Imaging; TR, repetition time; TE, echo time; FoV, field of view; TI, inversion time; PE, Phase Encoding.

For the WH study, two MRI scanners were used, due to the scanner upgrade two-thirds of the way through the study: a 3T Siemens Magnetom Verio scanner (SC1) with a 32-channel receive head coil (April 2012–December 2014) and a 3T Siemens Prisma scanner (SC2) with a 64-channel receive head-neck coil in the same centre (July 2015–December 2016). The MRI modalities used for WMH segmentation were Fluid Attenuated Inversion Recovery (FLAIR) scans, T1-weighed scans and diffusion-weighted scans (dMRI), to derive Fractional Anisotropy (FA) maps. The MRI sequence parameters were either identical or closely matched between the two scanners.

For the UKB dataset, MRI acquisition was carried out using a 3T Siemens Skyra with a 32-channel receive head coil (full details in Miller et al., 2016). As regards the MRI modalities, for the current study we used FLAIR scans and T1-weighed scans. We decided not to include dMRI within the WMH quantification pipeline, because the requirement to have 3 usable MRI modalities for each subject would have caused the exclusion of a small, yet significant amount of data (see Alfaro-Almagro et al., 2018 for an indication of usable data for each modality). In fact, currently released measures of WMHs for UKB are extracted using T1-weighted and FLAIR only. Moreover, unlike T1-weighted and FLAIR scans, dMRI with 6 or more directions (needed to perform Diffusion Tensor Imaging and generate FA maps) are not very common in clinical contexts. Therefore, being able to obtain consistent WMH estimates with common sequences would make our approach more widely applicable.

*MRI pre-processing* – All the available MRI scans underwent pre-processing using FSL v.6.0 tools (Jenkinson et al., 2012) before being fed to BIANCA for WMH segmentation. T1-weighted scans were processed using fsl_anat, which performs bias correction, brain extraction, and partial-volume tissue segmentation using FAST (Zhang et al., 2001). The sum of the volumes for the three tissue classes was used as total brain volume to normalise WMH measures. We used an exclusion mask for cortical grey matter and structures that can appear hyperintense on FLAIR and for which BIANCA is not currently optimised (details in Griffanti et al., 2016). FLAIR images were brain-extracted using BET (Smith, 2002) and bias field corrected with FAST (Zhang et al., 2001). Images without bias field correction were also used to evaluate the effect of this pre-processing step on the WMH measures. For WH data, dMRI scans were pre-processed as described in (Filippini et al., 2014) and a diffusion tensor model was fit at each voxel to obtain FA maps.

Since BIANCA works in single-subject space, we used FLIRT (Jenkinson and Smith, 2001) to register all the MRI modalities to the FLAIR scan, chosen as reference modality. Then, we masked the latter with the exclusion mask derived from the T1-weighted images. The transformation between FLAIR and MNI space for each subject was also calculated (using FLIRT) to be used by BIANCA to derive the spatial features (MNI coordinates).

As BIANCA requires several parameter choices, we tested the influence of those that are particularly relevant for harmonisation, while keeping the others constant. We performed a preliminary analysis to assess the best combination of settings that produced consistent performances for segmentation accuracy and specificity across datasets. The best settings were found to be in line with the suggested parameters in (Griffanti et al., 2016) and previously used in studies using BIANCA on the WH dataset (Griffanti et al., 2018). Therefore, we fixed the following parameters for BIANCA throughout our study: 2000 training points representing WMH lesions, 10,000 points representing non-lesion voxels, a patch size of dimension 3 voxels and a spatial weighting coefficient equal to 2. The number of k nearest neighbours used in the algorithm was set to *k* = 40, since it provided good performance in previous studies using k-NN for white matter lesion segmentation (Anbeek et al., 2004; Steenwijk et al., 2013; Griffanti et al., 2016).

A subset of manually segmented WMH images was available from each dataset to train BIANCA and to evaluate its segmentation performance in a cross-validated manner. The segmented data included 24 participants from the WH1 dataset, 24 from the WH2 dataset and 12 from the UKB dataset. The 24 subjects from WH1 were manually annotated by two raters (R1, R2). Rater 2 repeated their annotation a year later (R2a, R2b) enabling us to assess the effects of within- and between-rater variability on the WMH measures. Rater 2 also labelled the 24 scans from the WH2 dataset. For UKB we used the manual masks of 12 subjects used in the released imaging pipeline (Alfaro-Almagro et al., 2018).

### 2.2 Harmonisation pipeline

During our work we dealt with two scenarios: the first aimed to harmonise the two Whitehall imaging sub-studies (WH1 and WH2) representing data before and after the scanner upgrade within the same cohort and centre; the second addressed the integration of the WH and UKB cohorts, which were acquired on different scanners at different centres. The two scenarios allowed us to test the effect of different factors affecting data and required some changes in the harmonisation pipelines applied.

*Scanner upgrade (Whitehall)* – We started the analysis with the scanner upgrade scenario (WH1 and WH2) that included prospective harmonisation in the study design: the same non-imaging variables were collected and the MRI protocol was as close as possible for the two scanners. Retrospective harmonisation was therefore not needed for the non-imaging data but carried out on the images.

The availability of manual masks from multiple raters, ‘traveling heads’ data and FA maps for most of the participants allowed us to study the impact of: (i) the rater performing the manual labelling, (ii) the process of bias field correction on FLAIR images, (iii) the composition of the dataset used to train BIANCA (training set) and (iv) the inclusion of FA as one of the MRI modalities used by the segmentation tool to derive intensity features. We compared one option at a time using the metrics described in the Evaluation metrics section, while keeping the others fixed, in order to understand how each one could influence the results. We then identified optimal pre-processing and analysis strategies to reduce non-biological variability across datasets, while retaining or taking into account (modelling) the biological variability.

**Effect of rater**: in the training phase, BIANCA requires manually delineated WMH masks, which are known to suffer from inter- and intra-rater variability (Guo et al., 2019). We wanted to assess whether BIANCA trained with different manual masks (either multiple annotations by different raters or repeated annotations by the same rater) generates WMH segmentations that are more or less variable than the manual annotations among themselves. If BIANCA produced more consistent WMH masks than manual operators, the use of this automated segmentation tool would be advisable to obtain more consistent results. We evaluated this on data from a single scanner (WH1). We had multiple annotations for 24 MRI scans (two raters - R1, R2; and two annotations by R2 one year apart - R2a, R2b - corresponding manual masks M1, M2a, M2b). Between-rater (M1 vs M2a; M1 vs M2b) and within-rater (M2a vs M2b) agreement was calculated in terms of overlap between the manual masks using Dice Similarity Index (DI – see Griffanti et al., 2016). Each set of ratings was then used to train BIANCA and the automated WMH masks (B1, B2a, B2b) were generated using a leave-one-out approach. We then calculated between-rater (B1 vs B2a; B1 vs B2b) and within-rater (B2a vs B2b) agreement also on the automatically segmented masks using DI. Finally, we compared DI values using paired t-tests to assess whether consistency within the automatic WMH segmentations was higher or lower with respect to consistency within the manually labelled masks.

**Effect of bias field correction**: we assessed the impact of bias field correction (BC) in multiple ways. One indication of successful harmonisation is that harmonised images should be more similar to each other. We evaluated this aspect on the ‘traveling heads’ data available for the WH dataset. Corresponding scans from each of the 5 subjects were first registered to each other and then resampled into the space half-way between the two. We then calculated the cost function (correlation ratio) between the registered images as a measure of image similarity that is not influenced by head position (lower cost function indicates more similar images). The same procedure was repeated on the bias field corrected images. The values of the cost function before and after BC were compared with a paired *t*-test. Secondly, we investigated the effect of BC on BIANCA performance (i.e. overlap with manual WMH masks) as described in the Evaluation metrics section. The manual rater was R2 for both datasets and the training set for BIANCA was the same (24 subjects from WH1). We compared the results obtained before and after BC, to test whether the adoption of this pre-processing step could provide more consistent results across datasets. We then evaluated the effect of BC on the relationship between WMHs and age, and in terms of explained variability of the scanner effect in a multivariate regression model (see Evaluation metrics for details).

**Effect of training set composition for BIANCA**: we compared three options that could be used to train BIANCA when performing WMH segmentation on multiple datasets: single-site training (using the same training set for all datasets, with examples coming only from one site - 24 subjects from WH1 in our case), site-specific training (training BIANCA on each dataset separately) and mixed training (combining examples from WH1 and WH2, 24 subjects each, in a single training set to apply to all datasets). As before, we exploited several analysis approaches to evaluate which option would lead to better harmonised WMH measures. We investigated the effect of each option on: BIANCA performance, the relationship between WMHs and age, and the weight of the scanner variable in the multivariate regression model. All data were bias field corrected before the analysis (see Evaluation metrics for details).

**Effect of FA information**: as previously mentioned, we did not use FA maps derived from dMRI to inform WMH segmentation for the UKB dataset, but FA maps were used in the WH dataset. Aiming to ultimately integrate the two datasets, we assessed on WH datasets the impact of not using FA as an additional intensity feature for BIANCA. We compared the FA inclusion/exclusion cases in terms of BIANCA performance, relationship between WMHs and age, and the weight of the scanner variable in the multivariate regression model (see Evaluation metrics for details). For testing this option, we only used bias field corrected images and fixed BIANCA training set to be mixed (i.e. including examples from WH1 and WH2).

*Retrospective harmonisation of Whitehall and UK Biobank datasets* – We then extended the investigation to include data from the UKB cohort. In this case, no prospective harmonisation had been performed for imaging or non-imaging variables. The cohorts, despite being ageing populations, differ in many aspects (see Table 1 for details). Hence, both non-imaging and imaging data required harmonisation.

**Non-imaging harmonisation**: non-imaging data available for both WH and UKB were converted to a common format. The conversion was conducted using the FMRIB UKBiobank Normalisation, Parsing And Cleaning Kit (FUNPACK) (McCarthy, 2019), a Python library for pre-processing of UKB data containing a large number of procedures allowing us to perform various data sanitisation and processing steps. We defined a configuration file for FUNPACK, currently available online on GitLab (https://issues.dpuk.org/eugeneduff/wmh_harmonisation). It includes both built-in rules and new conversion functions that allowed us to obtain non-imaging variables expressed in the same units of measurements.

**Imaging data harmonisation – effect of training set composition for BIANCA**: for WH-UKB integration, the manual WMH masks were generated by different raters, bias field correction was already performed as part of the automated pre-processing pipeline (Alfaro-Almagro et al., 2018) and FA was not used as additional intensity feature. We therefore tested whether the use of a specific training set for BIANCA could improve harmonisation between UKB and WH, despite different raters providing WMH examples and the use of only T1 and FLAIR as intensity features. Similar to the previous scenario, we compared the impact of site-specific and mixed training sets (this time combining examples from WH1, WH2 and UBK). Also in this case, the evaluation included comparing BIANCA performance, the relationship between WMHs and age, and the weight of the scanner variable in the multivariate regression model (see Evaluation metrics for details).

*Evaluation metrics* – We evaluated the success of harmonisation in several ways.

First, the harmonised WMH segmentation pipeline should have the same (or as close as possible) WMH segmentation performance across datasets. To assess this, we calculated a series of overlap measures: Dice Similarity Index (DI), voxel-level False Positive Ratio (FPR), voxel-level False Negative Ratio (FNR), cluster-level FPR, cluster-level FNR (see Griffanti et al., 2016 for details) between manual WMH masks and automatically segmented WMH masks (obtained using leave-one-out cross-validation whenever appropriate). We matched the number and the approximate lesion load of the manually annotated scans used to evaluate the automatic segmentation performance for all datasets (12 subjects for each dataset, WH1, WH2, UKB). We then looked at how different these metrics were between datasets for each option tested (across-scanner evaluation within option). In the scanner upgrade scenario we compared metrics between SC1 and SC2 for each of the following options: (A) without BC, single-site training, FA included; (B) with BC, single-site training, FA included; (C) with BC, site-specific training, FA included; (D) with BC, mixed training, FA included; (E) with BC, mixed training, FA excluded. For the WH-UKB harmonisation we compared SC1 vs SC2 vs UKB for the (A) site-specific training and (B) mixed training options (both with BC and no FA).

Alongside the harmonisation aim, we also took into account the accuracy of the WMH segmentation (since consistent BIANCA performance across datasets does not necessarily correspond to accurate segmentation). Therefore – for each dataset – we compared BIANCA performance across different options ((A) vs (B) for bias field, (B) vs (C) vs (D) for training set, (D) vs (E) for effect of FA – for the scanner upgrade scenario; (A) vs (B) for training set – for the WH-UKB scenario) to investigate whether the adoption of one of them could lead to substantial improvements in terms of either segmentation accuracy, sensitivity or specificity (within-scanner evaluation across options).

When the number of available options for both the across- and within-subject factors (being dataset and analysis option, respectively) was equal to two (as for the rater, bias field, and FA assessment) we used two-sample independent t-tests and paired t-tests for statistical assessment. When the number of available options was higher than two (as for the training set assessment) we first performed a two-way mixed ANOVA test, to test for potential interaction between factors and then, if results were significant, we investigated the main effect of each factor through separate one-way ANOVA tests.

We then extended the evaluation to the full sample by considering the output of the automatic WMH segmentation for all the available subjects (WH1=513, WH2=200, UKB=2285), instead of just for those with manual WMH mask. We calculated WMH volumes (expressed as % of total brain volume) and compared them across datasets for each option of the two scenarios. In doing this we wanted to take sources of biological variability into account. Given that age is known to be among the strongest risk factors for WMHs, we started by looking at the correlation between WMH volumes and age in our datasets. We used a one-way ANCOVA test, with WMH volumes as the dependent variable, age as the main covariate and scanner/site as the categorical factor. Age was demeaned to avoid multicollinearity and make results more interpretable. With this test we assessed differences in terms of slope (interaction between age and scanner) and intercept at mean age (main effect of scanner) for each option. Similar regression slopes (no significant interaction) and reduced or no volume bias (no significant main effect of scanner) would indicate successful harmonisation. When the hypothesis of homogeneous regression slopes was not met (i.e. when slopes were significantly different), we used the Johnson–Neyman technique to identify the “region of non-significance”, i.e. the range of age values for which there are no significant differences in WMH% between scanners (White, 2003).

Finally, harmonisation was evaluated by the extent to which it reduced the variation in WMH volumes that could be explained by scanner and dataset. We assessed this by examining the fit of a linear multivariate model, estimated using Elastic Net to reduce over-fitting (Pedregosa et al., 2011), that predicted WMH volumes from non-imaging variables (see Table 1 for details) (including a variable associated with scanner/dataset). Well harmonised datasets will have minimal variance attributed to the scanner/dataset variables. While non-linearities are likely to be present in the data, this linear approach allowed us to compare the effect of the different processing approaches in a highly interpretable way.

## 3 Results

### 3.1 Scanner upgrade (Whitehall)

**Fig. 1:**
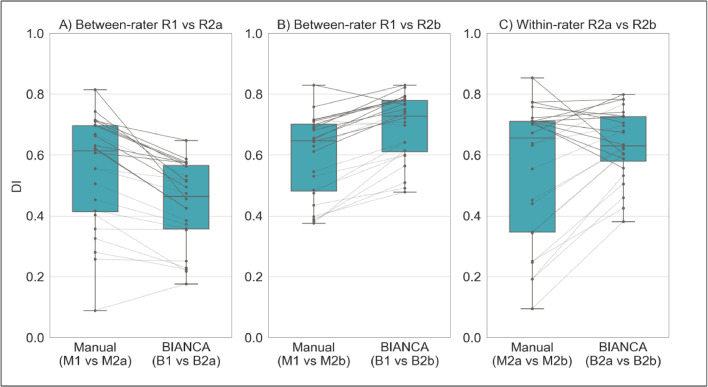
Effect of rater, assessed both in terms of between- (A and B) and within-rater variability (C). Each panel displays a comparison of the agreement (measured with Dice Similarity Index) between manual masks annotated by the raters (left box-plots) and BIANCA outputs generated with masks from those raters (right box-plot). Solid and dotted lines refer to results obtained on subjects characterised, respectively, by high and low WMH load. Legend: R1 = rater 1, R2a = Rater 2, first rating, R2b = rater 2, second rating (1 year apart from the first rating, blind to first rating), *M* = manual, *B* = BIANCA.

**Effect of rater.** Overall, BIANCA produced more consistent WMH masks than manual operators (Fig. 1). Comparing manual and automatic segmentation procedures in terms of between-rater variability (R1 vs R2), we obtained opposite results when considering either the first (R2a) or second rating (R2b) from the second rater. The comparison between R1 and R2a highlighted a higher agreement (higher DI values) between manual masks (M1 vs M2a) than between the corresponding BIANCA output (B1 vs B2a) (Fig. 1.A, *p* < 0.001 paired *t*-test). On the other hand, the comparison between R1 and R2b showed better consistency for BIANCA results (B1 vs B2b) than manual annotations (M1 vs M2b) (Fig. 1.B, *p* < 0.001 paired *t*-test). It is worth noting that the worst agreements (both between manual masks and BIANCA outputs) were observed for subjects characterised by very low WMH loads (dotted lines). For within-rater (R2) variability, we observed that BIANCA outputs (B2a vs B2b) had higher consistency than manual masks annotated twice by the same operator (M2a vs M2b) (Fig. 1.C, *p* < 0.001 paired *t*-test). For full details of the DI statistics across these comparisons refer to Supplementary Table S2.

**Fig. 2:**
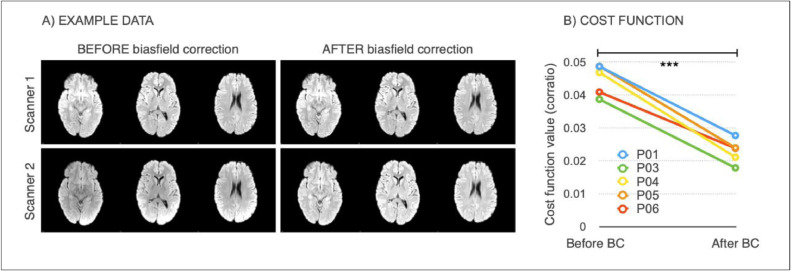
Effect of bias field correction (BC) on ‘travelling heads’ data from the WH dataset. (A) example data from 1 subject acquired on both scanners, before and after BC showing improvement in image similarity after BC (B) Cost function (correlation ratio) between Scanner1/Scanner2 images of the 5 traveling head participants, calculated before and after BC (*** - *p* < 0.001).

**Effect of bias field correction.** BC led to increased image similarity, when comparing ‘traveling heads’ data from the two WH scanners, as clearly visible from the example shown in Fig. 2.A. This was confirmed by a significant decrease in the cost function (correlation-ratio) after BC (*p* < 0.001 paired *t*-test; Fig. 2.B).

**Fig. 3:**
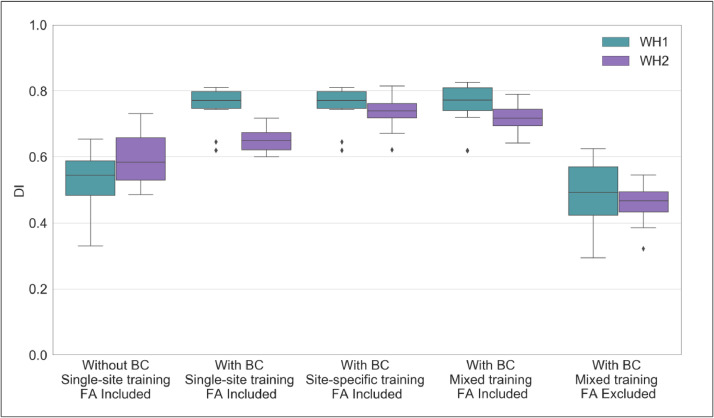
BIANCA performance – scanner upgrade scenario. Box-plot of the Dice Similarity Index (DI) between BIANCA output and the corresponding manual masks for the different analysis options tested during our study (specified on the *x* axis). All the displayed results were evaluated on a sub-sample of manually segmented subjects (12 for WH1 and 12 for WH2) balanced in terms of WMH load and using leave-one-out cross-validation whenever appropriate (details in the main text).

The effects of bias field correction on BIANCA performance are shown in Table 3 and Fig. 3 (where Fig. 3 displays DI values, while the equivalent plots for the other metrics are reported in the Supplementary material). Comparing segmentation performance within scanner, we observed a significant increase in the overall segmentation accuracy after BC, with higher DI values for both WH1 and WH2 datasets (Fig. 3). Moreover, BC led to a greater level of specificity for the WH2 dataset, demonstrated by a significant decrease in FPR and cluster-level FPR. For the WH1 dataset, the DI improvement was accompanied by a decrease of FNR and cluster-level FNR values. This was at the expense of an increase in the WH1 FPR and cluster-level FPR. There was a significant difference in DI values between WH1 and WH2 after but not before BC. This was likely due to a combination of uneven increase of accuracy in WH1 and WH2 and a reduction of the variability of DI values within scanner (smaller interquartile range in boxplots – Fig. 3). However, BC also had a positive impact on FPR which were no longer significantly different across-scanners.

**Table 3:** BIANCA performance – scanner upgrade scenario – Summary of all the overlap measures between BIANCA output and the corresponding manual mask, calculated for the different analysis options tested in our study (using leave-one-out cross-validation whenever appropriate). Statistical tests performed on data to assess the impact of bias field correction, training modalities and FA inclusion/exclusion on the segmentation performance.

			DI		FPR		FNR		cluster-level FPR		cluster-level FNR	
			WH1	WH2	WH1	WH2	WH1	WH2	WH1	WH2	WH1	WH2
Overlap measures	Mean ± std	Option A	0.52 ± 0.10	0.59 ± 0.07	0.05 ± 0.04	0.33 ± 0.16	0.63 ± 0.09	0.43 ± 0.08	0.09 ± 0.07	0.69 ± 0.14	0.610.13	0.48 ± 0.11
		Option B	0.75 ± 0.06	0.64 ± 0.03	0.18 ± 0.08	0.22 ± 0.10	0.28 ± 0.09	0.42 ± 0.07	0.33 ± 0.17	0.57 ± 0.18	0.35 ± 0.17	0.47 ± 0.09
		Option C	0.75 ± 0.06	0.73 ± 0.05	0.18 ± 0.08	0.26 ± 0.12	0.28 ± 0.09	0.24 ± 0.08	0.33 ± 0.17	0.53 ± 0.18	0.35 ± 0.17	0.35 ± 0.12
		Option D	0.76 ± 0.05	0.71 ± 0.04	0.22 ± 0.09	0.23 ± 0.11	0.23 ± 0.08	0.30 ± 0.07	0.42 ± 0.16	0.52 ± 0.17	0.28 ± 0.15	0.40 ± 0.10
		Option E	0.48 ± 0.11	0.45 ± 0.06	0.07 ± 0.05	0.09 ± 0.09	0.66 ± 0.09	0.68 ± 0.06	0.15 ± 0.09	0.17 ± 0.10	0.55 ± 0.14	0.63 ± 0.10
Effect of Bias field correction	Between-subject analysis: independent t-test		WH1 vs WH2		WH1 vs WH2		WH1 vs WH2		WH1 vs WH2		WH1 vs WH2	
		Option A	0.061		< 0.001 ***		< 0.001 ***		< 0.001 ***		0.020 *	
		Option B	< 0.001 ***		0.259		< 0.001 ***		0.004 **		0.049 *	
	Within-subject analysis: paired t-test	Option A vs Option B	< 0.001 ***	0.035 *	< 0.001 ***	0.002 **	< 0.001 ***	0.531	< 0.001 ***	< 0.001 ***	< 0.001 ***	0.306
Effect of Training modalities	Training - Scanner interaction: two-ways mixed ANOVA test		< 0.001 ***		< 0.001 ***		< 0.001 ***		< 0.001 ***		< 0.001 ***	
	Main effect of the Scanner (between-subject factor): independent t-test		WH1 vs WH2		WH1 vs WH2		WH1 vs WH2		WH1 vs WH2		WH1 vs WH2	
		Option B	< 0.001 ***		0.259		< 0.001 ***		0.004 **		0.049 *	
		Option C	0.433		0.071		0.272		0.013 **		0.998	
		Option D	0.046 *		0.861		0.049 *		0.178		0.049 *	
	Main effect of the Training (within-subject factor): repeated measures one-way ANOVA test (F-test and post-hocs)		0.466	< 0.001 ***	< 0.001 ***	< 0.001 ***	< 0.001 ***	< 0.001 ***	< 0.001 ***	0.036 *	< 0.001 ***	< 0.001 ***
		Option B vs Option C	———	< 0.001 ***	———	< 0.001 ***	———	< 0.001 ***	———	0.45	———	< 0.001 ***
		Option B vs Option D	———	< 0.001 ***	< 0.001 ***	0.309	< 0.001 ***	< 0.001 ***	< 0.001 ***	0.045 *	< 0.001 ***	< 0.001 ***
		Option C vs Option D	———	0.044 *	< 0.001 ***	< 0.001 ***	< 0.001 ***	< 0.001 ***	< 0.001 ***	1	< 0.001 ***	0.002 **
Effect of FA inclusion/ exclusion	Between-subject analysis: independent t-test		WH1 vs WH2		WH1 vs WH2		WH1 vs WH2		WH1 vs WH2		WH1 vs WH2	
		Option D	0.046 *		0.861		0.049 *		0.178		0.049 *	
		Option E	0.462		0.461		0.484		0.565		0.134	
	Within-subject analysis: paired t-test	Option D vs Option E	< 0.001 ***		< 0.001 ***		< 0.001 ***		< 0.001 ***		< 0.001 ***	

Options tested in our study are: (A) without BC, single-site training, FA included; (B) with BC, single-site training, FA included; (C) with BC, site-specific training, FA included; (D) with BC, mixed training, FA included; (E) with BC, mixed training, FA excluded. For each metric we reported: (i) mean ± std values relative to all datasets involved in our study (WH1, WH2); (ii) impact exerted by bias field correction on BIANCA performance (between- and within-subject analysis performed using independent and paired t-tests respectively); (iii) impact exerted by training modalities on BIANCA performance (two-ways mixed ANOVA test assessing the interaction between training and scanner (between- and within-subject factors respectively); when the interaction term resulted being significant we decomposed the analysis in two separate components assessing the main effect of training (repeated measures one-way ANOVA test evaluating differences between the investigated options for each dataset involved in our study; F-test and post-hoc comparisons are displayed) and the main effect of scanner (independent t-test evaluating differences between the investigated dataset for each option involved in our analysis); (iv) impact exerted by FA inclusion/exclusion on BIANCA performance (between- and within-subject analysis performed using independent and paired t-tests respectively). Results relative to the statistical tests are all reported in terms of *p*-values: * (< 0.05), ** (< 0.01), *** (< 0.001). Legend: DI = Dice Similarity Index, FPR = False Positive Ratio, FNR = False Negative Ratio, WH1 = Whitehall dataset 1, WH2 = Whitehall dataset 2.

**Fig. 4:**
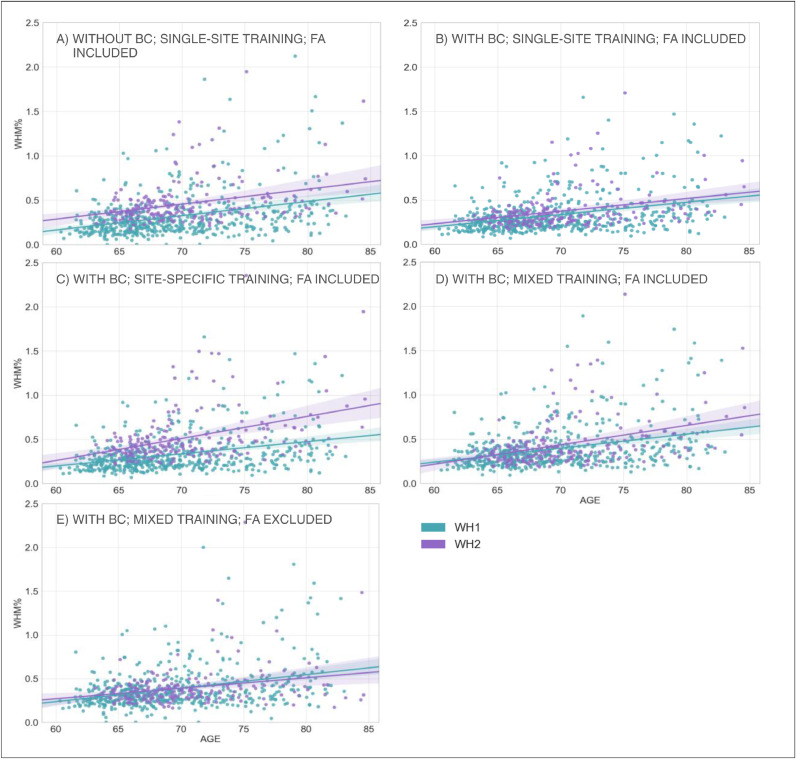
Association between WMHs and age – scanner upgrade scenario. Scatter plot of the relationship between WMH volumes (expressed as % of total brain volume, *y* axis) and age (*x* axis), for WH1 (cyan) and WH2 (purple) data. Regression lines with 95% confidence interval are also displayed. Each plot refers to one of the investigated analysis options: (A) without BC, single-site training, FA included; (B) with BC, single-site training, FA included; (C) with BC, site-specific training, FA included; (D) with BC, mixed training, FA included; (E) with BC, mixed training, FA excluded. Evaluation was conducted on the full sample of data for both datasets (WH1 = 513, WH2 = 200) (For interpretation of the references to color in this figure legend, the reader is referred to the web version of this article.).

We then analysed the correlation between WMH volumes and age to determine the extent to which this relationship was affected by the scanner for the two BC options (Fig. 4.A and B). Results of the one-way ANCOVA tests reported in Table 5 show no significant difference when comparing regressions slopes between scanners for both options (*p*-value=0.836 before BC; *p*-value=0.892 after BC). A significant across-scanner difference was instead found in the intercepts – in correspondence to the mean age – both before and after BC. However, the difference was reduced after BC (*p*-value<0.001 before BC; *p*-value=0.029 after BC).

**Fig. 5:**
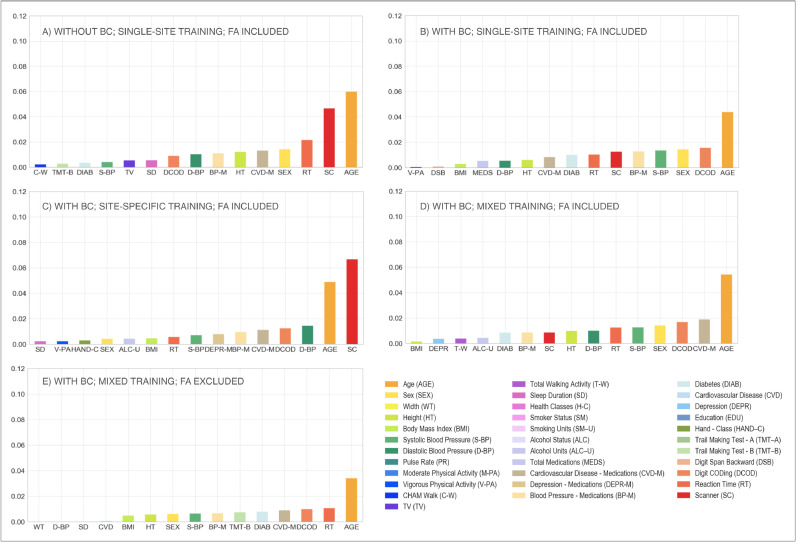
Multivariate model – scanner upgrade scenario. Percentage of variance (*y* axis) explained by non-imaging variables (reported on the *x* axis) in the linear multivariate model that was implemented (Elastic Net). Evaluation was conducted on the full sample of data (WH1 = 513, WH2 = 200). Each plot refers to one of the investigated analysis options: (A) without BC, single-site training, FA included; (B) with BC, single-site training, FA included; (C) with BC, site-specific training, FA included; (D) with BC, mixed training, FA included; (E) with BC, mixed training, FA excluded. Variable scanner/site (SC) highlighted in red. Values are reported in Table 6 and Supplementary Table S3 (For interpretation of the references to color in this figure legend, the reader is referred to the web version of this article.).

Finally, the implemented Elastic Net model showed that, after BC, the amount of variance in WMH volume attributed to the scanner/site of acquisition was lower (from 0.046 to 0.012, see Table 6), passing from second to sixth position (Fig. 5.A and B). Elastic Nets were used to reduce the likelihood of over-fitting of these complex models. While regularisation may in some cases have unpredictable effects on regression parameters, in this case matching results were observed when models were fit using standard Ordinary Least Squares (OLS).

**Effect of training set composition for BIANCA.** Overall, our results suggest that the mixed-training option offers the best trade-off among the explored evaluation metrics, providing good and consistent BIANCA performance and consistent WMH volumes.

When investigating the presence of a significant interaction between scanners (WH1/WH2) and training options (single-site/site-specific/mixed), a two-way mixed ANOVA test gave significant results for all the assessed overlap measures (Table 3). Therefore, we investigated the effect of each factor separately, evaluating firstly across-scanner and then within-scanner performances. Site-specific training produced the most consistent segmentations with respect to across-scanner performance. Between the remaining two options, the mixed training showed better consistency with respect to single-site training, with no significant WH1-WH2 difference in the cluster-level FPR values. When comparing segmentation performance within-scanner, we observed an overall improvement of results in WH2 when BIANCA was trained using annotated data from WH2 (site-specific training) rather than from WH1 (single-site training). The significant improvements in DI, FNR and cluster-level FNR were only at the expense of increased FP values. The comparison between site-specific and mixed training led to different results for the two scanners, with significantly worse FPR and cluster-level FPR values for mixed training in WH1 and worse DI, FNR and cluster-level FNR values in WH2. The remaining indicators showed improved or unaltered performance with mixed training. Results showed a favourable pattern towards mixed over single-site training. For WH1, we observed a significant improvement for both FNR and cluster-level FNR when using mixed training, no significant difference in DI, and worse FPR and cluster-level FPR. For WH2, better performances were observed using a mixed training for all the indicators except FPR, which was not significantly different from the single-site training case.

The results obtained from the one-way ANCOVA tests (Table 5) showed that site-specific training led to a significant difference between the age regression slopes for the two scanners (*p*-value=0.004). The Johnson–Neyman technique further confirmed that using this option led to the highest differences in WMH% between scanners (Fig. 4.B, C and D) as the region of non-significance between WH1 and WH2 would have been between 51.13 and 57.95 years, a narrow range of values outside the age range of WH data (60–85 years). The adoption of a mixed training had a positive impact on regression slopes, such that they were no longer significantly different (*p*-value=0.129) and also reduced the volume bias (at the mean age) (*p*-value=0.052).

When site-specific training was used, the weight of the scanner/site variable was greatly increased in the multivariate regression model, compared to the single-site option (Fig. 5.B and C), with scanner/site being the variable that explained the greatest amount of variance (from 0.012 to 0.066, see Table 6). The adoption of a mixed training instead, reduced the amount of variance explained by the scanner/site variable (from 0.066 to 0.008, see Table 6), with the variable moving to the ninth position (Fig. 5.D).

**Effect of FA information.** The removal of FA as an additional intensity feature for WMH segmentation led to higher consistency between sites, but lower segmentation accuracy.

Without FA there were no significant differences between the WH1 and WH2 datasets in all performance metrics. There was a significant decrease in the overall segmentation accuracy when excluding FA from the intensity features used by BIANCA, with lower DI performances (Fig. 3), and a negative impact on both FNR and cluster-level FNR (worse sensitivity). Removing FA also lowered FPR and cluster-level FPR, leading to a greater level of specificity.

For the correlation between WMH volumes and age, results of the one-way ANCOVA tests (Table 5) showed that, excluding FA, the difference in slopes remained not significant (*p*-value=0.379). The already small volume bias (at mean age) was further decreased (*p*-value=0.874) (Fig. 4.D and E).

Extracting WMHs using FLAIR and T1-weighted images only led to a decrease in the variance explained by the scanner/site variable (form 0.008 to 0.000, see Table 6), which was no longer present amongst the most predictive features (Fig. 5.D and E).

*Retrospective harmonisation of Whitehall and UK Biobank datasets*

**Non-imaging harmonisation.** By applying our configuration file for FUNPACK, we brought all the variables into the same units for both datasets. Table 1 shows the format/units that each of the selected non-imaging variables were originally acquired with in WH and UKB, as well as the harmonised units chosen and the resulting harmonised mean and standard deviation values.

**Imaging data harmonisation – effect of training set composition for BIANCA.** We next assessed the impact of different training sets (site-specific and mixed training) on the level of harmonisation between the WH and UKB WMH datasets (the single-site training was not tested, as it gave poor results in the scanner upgrade scenario).

**Fig. 6:**
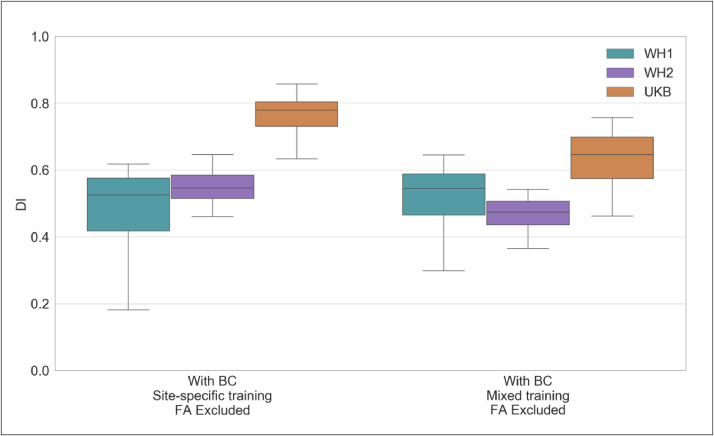
BIANCA performance – retrospective data merging scenario. Box-plot of the Dice Similarity Index (DI) between BIANCA output and the corresponding manual mask for the different analysis options tested during our study (specified on the *x* axis) All the displayed results were evaluated on a sub-sample of manually segmented subjects (12 for WH1, 12 for WH2 and 12 for UKB) balanced in terms of WMH load and using leave-one-out cross-validation.

Results for BIANCA performance in terms of Dice Similarity Index (DI) are shown in Fig. 6. The equivalent plots for the other metrics are reported in the Supplementary material. The two-way mixed ANOVA test highlighted the presence of a significant interaction between the scanners (WH1/WH2/UKB) and the training options (site-specific/mixed) for all the overlap measures (Table 4). For this reason, we further evaluated the main effect of each factor, investigating across- and within- scanner performance separately. Results of the one-way ANOVA test revealed significant differences for all metrics across scanners when using a mixed training. The site-specific training gave more homogeneous results, (non-significant FPR and cluster-level FPR). Post-hoc pairwise comparisons revealed no significant difference in any overlap metrics between WH1 and WH2 for either of the training options. On the other hand, UKB showed a different performance with respect to the other datasets (WH1, WH2), using either site-specific or mixed training. Significant differences between WH1 and UKB were observed in DI, FNR and cluster-level FNR in the site-specific training case. DI, FNR and cluster-level FPR were significantly different in the mixed training case. All overlap metrics were significantly different between WH2 and UKB, except FPR and cluster-level FPR using site-specific training. Within-scanner comparisons highlighted a more favourable pattern towards the site-specific training. In fact, the use of a mixed training dataset led to improved segmentation sensitivity only for WH1, with a significant decrease of cluster-level FNR, and improved specificity for UKB with lower FPR and cluster-level FPR.

**Table 4:** BIANCA performance – retrospective data merging scenario – Summary of all the overlap measures between BIANCA output and the corresponding manual mask, calculated for the different analysis options tested in our study (using leave-one-out cross-validation). Statistical tests performed on data to assess the impact of training modalities on the segmentation performance.

			DI			FPR			FNR			cluster-level FPR			cluster-level FNR		
			WH1	WH2	UKB	WH1	WH2	UKB	WH1	WH2	UKB	WH1	WH2	UKB	WH1	WH2	UKB
Overlap measures	Mean ± std	Option A	0.47 ± 0.12	0.55 ± 0.06	0.76 ± 0.07	0.05 ± 0.04	0.10 ± 0.08	0.07 ± 0.04	0.67 ± 0.10	0.58 ± 0.08	0.34 ± 0.09	0.10 ± 0.08	0.20 ± 0.14	0.15 ± 0.12	0.61 ± 0.13	0.57 ± 0.11	0.41 ± 0.16
		Option B	0.51 ± 0.10	0.46 ± 0.05	0.62 ± 0.09	0.08 ± 0.05	0.10 ± 0.09	0.02 ± 0.01	0.63 ± 0.09	0.68 ± 0.05	0.52 ± 0.10	0.17 ± 0.11	0.18 ± 0.09	0.07 ± 0.07	0.54 ± 0.14	0.62 ± 0.10	0.45 ± 0.16
Effect of Training	Training - Scanner interaction: two-ways mixed ANOVA test		< 0.001 ***			< 0.001 ***			< 0.001 ***			< 0.001 ***			< 0.001 ***		
	Main effect of the Scanner (between-subject factor): one-way ANOVA test (F-test and post-hocs)	Option A		< 0.001 ***		0.199				< 0.001 ***		0.138				0.003 **	
			WH1 vs WH2	WH1 vs UKB	WH2 vs UKB	––––––––––––			WH1 vs WH2	WH1 vs UKB	WH2 vs UKB	––––––––––––			WH1 vs WH2	WH1 vs UKB	WH2 vs UKB
			0.122	< 0.001 ***	< 0.001 ***				0.155	< 0.001 ***	< 0.001 ***				0.835	0.004 **	0.019 *
		Option B	< 0.001 ***			0.027 *			< 0.001 ***			0.018 *			0.019 *		
			WH1 vs WH2	WH1 vs UKB	WH2 vs UKB	WH1 vs WH2	WH1 vs UKB	WH2 vs UKB	WH1 vs WH2	WH1 vs UKB	WH2 vs UKB	WH1 vs WH2	WH1 vs UKB	WH2 vs UKB	WH1 vs WH2	WH1 vs UKB	WH2 vs UKB
			0.343	0.009 **	< 0.001 ***	0.800	0.110	0.027 *	0.410	0.013 *	< 0.001 ***	0.987	0.043 *	0.030 *	0.318	0.298	0.014 *
	Main effect of the Training (within-subject factor): paired t-test	Option A vs Option B	0.255	< 0.001 ***	< 0.001 ***	< 0.001 ***	0.672	< 0.001 ***	0.171	< 0.001 ***	< 0.001 ***	0.008 **	0.304	0.010 *	< 0.001 ***	< 0.001 ***	< 0.001 ***

Options tested in our study are: (A) with BC, site-specific training, FA excluded; (B) with BC, mixed training, FA excluded. For each metric we reported: (i) mean ± std values relative to all datasets involved in our study (WH1, WH2, UKB); (ii) impact exerted by training modalities on BIANCA performance (two-ways mixed ANOVA test assessing the interaction between training and scanner (between- and within-subject factors respectively); when the interaction term resulted being significant we decomposed the analysis in two separate components assessing the main effect of training (paired *t*-tests evaluating differences between the investigated options for each dataset involved in our study) and the main effect of scanner (one-way ANOVA tests evaluating differences between the investigated dataset for each option involved in our analysis; F-test and post-hoc comparisons are displayed). Results relative to the statistical tests are all reported in terms of *p*-values: * (< 0.05), ** (< 0.01), *** (< 0.001). Legend: DI = Dice Similarity Index, FPR = False Positive Ratio, FNR = False Negative Ratio, WH1 = Whitehall dataset 1, WH2 = Whitehall dataset 2.

**Fig. 7:**
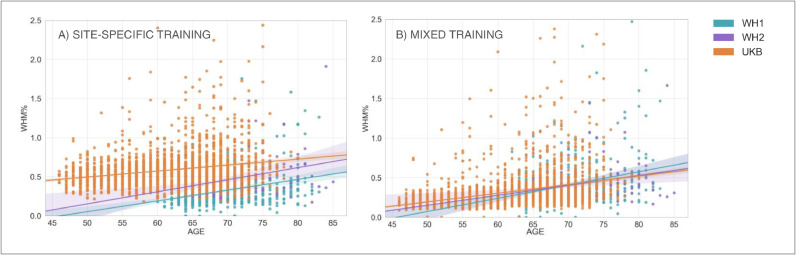
Association between WMHs and age – retrospective data merging scenario. Scatter plot of the relationship between WMH volumes (expressed as % of total brain volume, *y* axis) and age (*x* axis), for WH1 (cyan), WH2 (purple) and UKB (orange) data. Regression lines with 95% confidence interval are also displayed. Each plot refers to one of the investigated analysis options: (A) with BC, site-specific training, FA excluded; (B) with BC, mixed training, FA excluded. Evaluation was conducted on the full sample of data for all datasets (WH1 = 513, WH2 = 200, UKB = 2285) (For interpretation of the references to color in this figure legend, the reader is referred to the web version of this article.).

In terms of correlation between WMH volumes and age, we compared results for WH1, WH2 and UKB (Table 5). With respect to the site-specific case, the adoption of a mixed training led to a slight decrease in the across-scanner difference in regression slopes, although it still remained significant (*p*-value=0.004 mixed training; *p*-value=0.001 site-specific training, one-way ANCOVA test). However, as the hypothesis of homogeneous regression slopes was not met in either case, we used the Johnson–Neyman procedure to evaluate the WMH% differences between scanners. For the site-specific case there was no region of non-significance, since the three scanners were different across the whole range of age values. On the other hand, the use of a mixed training set led to a substantial decrease in the bias (Fig. 7.A and B) and the Johnson–Neyman region of non-significance (66.06–75.54 years) fell within the age range of our data (46–85 years).

**Table 5:** Analysis of the relationship between WMH volumes and age – scanner upgrade and retrospective data merging scenario – Summary of the one-way ANCOVA test and Johnson–Neyman (J-N) procedure.

	Scanner upgrade scenario					Retrospective data merging scenario	
	Analysis option A	Analysis option B	Analysis option C	Analysis option D	Analysis option E	Analysis option B	Analysis option C
One-way ANCOVA Slope	F(1, 709) = 0.043, *p* = 0.836	F(1, 709) = 0.019, *p* = 0.892	F(1, 709) = 8.358, *p* = 0.004 **	F(1, 709) = 2.303, *p* = 0.129	F(1, 709) = 0.774, *p* = 0.379	F(1, 2994) = 10.705, *p* = 0.001 **	F(1, 2994) = 8.334, *p* = 0.004 **
One-way ANCOVA Intercept	F(1, 709) = 43.678, *p* < 0.001 ***	F(1, 709) = 4.772, *p* = 0.029 *	—————————–	F(1, 709) = 3.789, *p* < 0.052	F(1, 709) = 0.025, *p* = 0.874	—————————–	—————————–
J-N Region of non-significance (age interval in years)	—————————–	—————————–	51.13–57.95	—————————–	—————————–	N/A	66.06–75.54

Options tested in our study are: (I) for the scanner upgrade scenario: (A) without BC, single-site training, FA included; (B) with BC, single-site training, FA included; (C) with BC, site-specific training, FA included; (D) with BC, mixed training, FA included; (E) with BC, mixed training, FA excluded; (II) for the retrospective data merging scenario: (A) with BC, site-specific training, FA excluded; (B) with BC, mixed training, FA excluded. The one-way ANCOVA test evaluated across-scanner differences (between WH1/WH2 or between WH1/WH2/UKB) characterising regression slope (interaction between age and scanner) and intercept at mean age (main effect of scanner) in the linear model relating WMH% to age. Results of the ANCOVA test are reported in terms of F(df)- and *p*-values: * (< 0.05), ** (< 0.01), *** (< 0.001). When the hypothesis of homogeneous regression slopes was not met, we used the Johnson–Neyman procedure to evaluate the across-scanner differences. Results for the Johnson–Neyman procedure are reported in terms of age intervals. Legend: N/A = Not Available (i.e. there was no age interval where WMH% were not different across scanners).

**Fig. 8:**
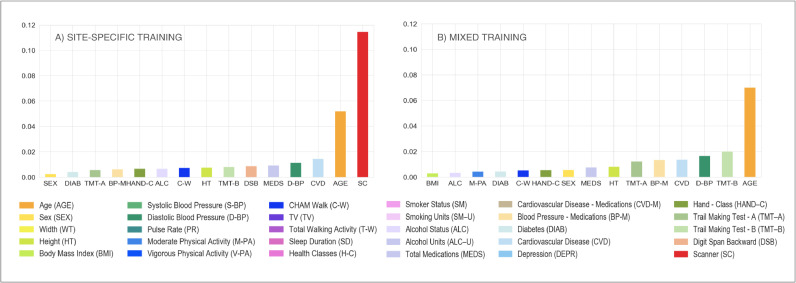
Multivariate model – retrospective data merging scenario. Percentage of variance (reported on the y axis) explained by non-imaging variables (reported on the x axis) in the linear multivariate model that was implemented (Elastic Net). Evaluation was conducted on the full sample of data for all the involved populations (WH1 = 513, WH2 = 200, UKB = 2285). Each plot refers to one of the investigated analysis options: (A) with BC, site-specific training, FA excluded; (B) with BC, mixed training, FA excluded. Variable scanner/site (SC) highlighted in red. Values are reported in Table 6 and Supplementary Table S3 (For interpretation of the references to color in this figure legend, the reader is referred to the web version of this article.).

The Elastic Net regression modelling showed that the scanner/site was no longer present amongst the most predictive features when using mixed training, compared to site-specific training (Fig. 8.A and B) where it explained the highest amount of variance in WMH volumes (from 0.115 to 0.000, see Table 6).

**Table 6:** Elastic Net Regression performance – retrospective data merging scenario – Summary of the results in terms of variance explained by the model and by age and scanner, the features considered most relevant in our study. Full details of all the other features are provided in Supplementary Table S3.

		Scanner upgrade scenario					Retrospective data merging scenario	
		Analysis option A	Analysis option B	Analysis option C	Analysis option D	Analysis option E	Analysis option A	Analysis option B
Variance explained by the model		0.243	0.161	0.207	0.173	0.125	0.190	0.098
Variance explained by the features	Age	0.060	0.043	0.048	0.054	0.034	0.052	0.070
	Scanner	0.046	0.012	0.066	0.008	0.000	0.115	0.000

Options tested in our study are: (I) for the scanner upgrade scenario: (A) without BC, single-site training, FA included; (B) with BC, single-site training, FA included; (C) with BC, site-specific training, FA included; (D) with BC, mixed training, FA included; (E) with BC, mixed training, FA excluded; (II) for the retrospective data merging scenario: (A) with BC, site-specific training, FA excluded; (B) with BC, mixed training, FA excluded. The amount of WMH variance explained by the model is calculated using the *R*-squared coefficient and reported in the first row. The amount of WMH variance explained by the features is reported in the rest of the table for the most relevant variables (age and scanner).

The same analyses were repeated on a subset of age-matched subjects from the three datasets to test if the different age range of the three datasets could have biased the results. The analyses led to very similar results, which are reported in the supplementary material.

Finally, we investigated how the information from the mixed training dataset was used by BIANCA. Details of analyses and results are reported in the supplementary material. Briefly, on a subset of subjects, for each point of the images we calculated the proportion of neighbours from the three different datasets used by the kNN classifier. We observed that, while the highest proportion of neighbours tended to come from the same site as the test subject (52.90% of the neighbours on average), in all cases there were neighbours from other sites (47.10% on average), confirming that BIANCA effectively used information from all datasets.

## 4 Discussion

In this work we explored several analysis strategies to harmonise measures of white matter hyperintensities (WMHs) of presumed vascular origin across large-scale datasets. The ability to combine different datasets will enable addressing important questions regarding the nature of WMHs and their prognostic value. We dealt with data from three scanners across two studies on healthy ageing. The study design allowed us to assess two different scenarios: a scanner upgrade (analogous scenario to a multi-centre study, involving a single population acquired with the same acquisition protocol on two MRI scanners) and a retrospective data merging (two distinct large populations acquired with different acquisition protocols on different MRI scanners). Each dataset included both imaging and non-imaging data that were exploited to develop harmonisation strategies and evaluate the results. We used an automated segmentation tool, BIANCA, to extract WMH measures from each imaging dataset and investigated the impact of different factors on the comparability of WMH measures: the rater performing manual segmentation of the examples used to train BIANCA, the process of bias field correction of the FLAIR images, the composition of the dataset used to train BIANCA (training set) and the inclusion/exclusion of FA as one of the intensity features. We investigated different processing strategies aiming to find the combination that led to the most consistent results across scanners or studies. We evaluated the success of each strategy looking for the best trade-off between consistency of BIANCA performance, segmentation accuracy and consistency of WMH volumes, after modelling the biological variability in the datasets (age and other non-imaging variables related to WMHs).

BIANCA needs to be trained by providing manual WMH segmentations, which are known to be affected by inter- and intra-rater variability (Guo et al., 2019). We wanted to assess how BIANCA would cope with this source of variability. On data from a single scanner (WH1) we observed that the consistency of the manual segmentation of the data has a major impact on the final BIANCA outputs. If the manual segmentations provided to BIANCA are sufficiently similar between raters/ratings, the automated tool improves the consistency of the output, providing better within- and between-rater agreement than the manual raters/ratings themselves. On the other hand, if the agreement between manual masks is low, BIANCA results can be even less consistent than manual masks. This prompts the need to standardise the definition of WMHs, especially in light of the fact that even if an increase in rating consistency is eventually achieved, this does not necessarily mean the obtained results are better in terms of accuracy. While for other segmentation tasks, e.g. hippocampus segmentation, clear protocols exist for manual labelling (Zandifar et al., 2018), there is no such protocol for WMHs. It is also worth noting that the lowest agreements (both between manual and automatic results) were observed for subjects characterised by a very low WMH load. In these images, WMHs are likely to be more difficult to segment because of their less obvious appearance or small size. Specific guidelines should therefore aim to clarify these sources of ambiguity. This analysis was limited by the relatively small number of ratings available and the range of expertise of the raters (R1 neuroimaging researcher, R2 medical student trained and supervised by an experienced neurologist). However, the scope of this evaluation was to explore how differences in manual ratings can impact a supervised segmentation method like BIANCA.

Correcting for bias field had a positive impact on almost all the metrics used for evaluation, indicating that, overall, its adoption contributes to successful harmonisation. We observed increased image similarity when comparing ‘traveling heads’ data from the WH scanners, showing a clear removal of scanner-related variability in the images, regardless of the difference in time of day for the acquisition. BIANCA performance improved after BC, although in terms of consistency of performance between scanners, an improvement was only observable when BC was combined with a different strategy for the composition of the training dataset, such as re-training BIANCA within each scanner or merging multiple examples from different scanners (Fig. 3). The successful removal of non-biological differences with BC was also evident when considering the correlation between WMH volumes and age, which showed that BC preserved the relationship with age (slopes not significantly different) while causing a decrease in the volume bias in correspondence of the mean age. The regression modelling using Elastic Nets confirmed the improved harmonisation with a significant decrease in the importance attributed to the scanner/site of acquisition (with similar results for OLS). Bias field correction of T2-weighted (and FLAIR) images is, however, not always included in pre-processing pipelines. In this work we specifically assessed the impact of BC on WMH segmentation and confirmed that it is beneficial to obtain more consistent image segmentation outputs across datasets.

The information provided by dMRI proved to be useful to obtain accurate WMH segmentation. When using FA maps as one of the intensity features for BIANCA, the performance within-scanner was higher than when using only T1-weighted and FLAIR images. However, when using only two modalities, all the overlap measures were more consistent across scanners, and the volume bias was reduced. Furthermore, the scanner was no longer a significant predictor of WMH volumes in the regression model. The decision regarding whether to use FA would therefore depend on the application. While for an accurate segmentation it is useful to include features from diffusion-weighted scans, it also constitutes an additional source of variability across datasets and scanners, leading to less harmonised WMH measures. dMRI data may be more sensitive to scanner and protocol changes than T1 and FLAIR due to the complexity of the sequence. Extra sources of variability in the measurements can be introduced by differences in the angular and spatial resolution, the number and distribution of diffusion gradient directions, the b-values, and other acquisition protocol parameters (Tax et al., 2019; Fortin et al., 2017). Several harmonisation strategies have been developed for dMRI including statistical data pooling techniques (Fortin et al., 2017), dictionary learning architectures (St‐Jean et al., 2020) and registration-based methods (Mirzaalian et al., 2016; Mirzaalian et al., 2018), but it remains an active area of research (Tax et al., 2019; Ning et al., 2019, 2020). Further work in this area should allow integration of DTI-derived measures in multimodal analyses such as ours, while maintaining good consistency of results. Another aspect to keep in mind is that FA might not always be available (while T2-FLAIR and T1 scans are more commonly acquired), preventing the integration of datasets (or participants within a dataset) that do not have all of them available and usable.

Regarding the choice of the composition of the training dataset for BIANCA we started by exploring three options in the scanner upgrade scenario. We compared the effect of using the same set for all the sites (single site), re-training BIANCA within-scanner (site-specific), or merging examples from different scanners (mixed). Single site training led to the biggest difference in BIANCA performance across datasets and a significant bias in the volumes (significantly different intercept at the mean age), although the relationship with age remained consistent (non-significant difference in regression slopes, highest amount of variance explained by age). On the other hand, the site-specific training provided the highest and most consistent BIANCA performance (overlap with manual masks on the subset of subjects with manual labels available) but led to the biggest difference in WMH volumes on the whole sample (significantly different slopes of the regression lines, Johnson–Neyman region of non-significance not within the age range of interest, highest amount of variance explained by the scanner variable). The results observed for the mixed training set suggest it represents the best trade-off between good and consistent BIANCA performance, and consistent WMH volumes. Although this could also be due to the fact that more images were used in the mixed training, similar results were observed when using the same number of images (12 from each scanner).

We further compared the best performing options (site-specific vs mixed) when harmonising WMH measures between WH and UKB. Using bias field corrected data and FLAIR and T1 as intensity features, the results were similar to the scanner upgrade scenario. While the segmentation performance was overall higher in the case of site-specific training, the most consistent results were those obtained with the mixed training set. To better understand these results we investigated how the information from the mixed datasets was used by BIANCA. In fact, even if the training points come from different datasets, only the (*k* = 40) neighbours are used in the classification of each point. We therefore confirmed that in the mixed training option, the neighbours used by the algorithm were indeed coming from a mixture of examples from the different datasets (see supplementary material for details). Moreover, we verified that our results were not driven by the significantly different age ranges of the three datasets by repeating our analyses on a subset of age-matched subjects and finding very similar results (see supplementary material for details). We still cannot exclude the possibility of some volume over/underestimation especially in the younger subjects. Future evaluations on additional samples of younger subjects and with manual masks will be important to further investigate this aspect.

The choice of the most suitable training set should hence be made depending on the application. When prioritising a more accurate WMH segmentation, a site-specific training is likely to give the best performance. When the aim is to compare or merge multiple datasets, a mixed training set is more appropriate.

Both of the optimal options identified above would require the effort of generating, or having access to, some manual masks and having to re-train BIANCA. Even if the numbers required are not high (12 images per dataset proved to be enough), this could still be an unfeasible option for some applications. The use of a single training set for multiple datasets would still be a valid option, but in light of our results, the recommendation would be to carefully check the segmentation accuracy and, when combining the resulting volumes, to consider the use of further strategies in the analyses to address potential biases (e.g. additional covariate in statistical analyses). The fact that including more examples from different datasets improved the results suggests that a promising solution would be to build a larger and more representative/generalisable training set, including examples from more scanners/datasets, that could be widely used. Towards this, we are publicly sharing our mixed training sets^1^. Future work on more datasets should assess if, with a sufficiently large set of examples, a single training set is general enough to be able to be successfully applied to new datasets.

The implemented multivariate linear regression approach suggested that major differences between datasets were removed with optimal pre-processing without direct harmonisation between datasets. However, direct harmonisation approaches such as ComBat (Fortin et al., 2018), which estimate corrections between datasets, applied either directly to T2 images, or to the output of BIANCA, could further enhance the harmonisation of these datasets. ComBat, specifically, is focused on small datasets, taking advantage of commonalities across “batches” (here, space) to improve estimation of site effects. While it may not provide substantial benefits for the large datasets analysed here, this could bring benefits to the harmonisation of smaller datasets. Approaches such as ComBat could also correct for multiplicative site effects, although it is not clear these are a major factor for WMHs. We expect that non-linear modelling of the non-imaging variables of interest would be valuable in this endeavour, as WMHs greatly increase in prevalence at later ages.

An important part of retrospective data merging was also the harmonisation of non-imaging variables. Modelling the biological variability is crucial to obtain imaging measurements that are well aligned across datasets. The ad-hoc configuration file we created for FUNPACK allowed us to obtain matched variables, with the same units across the WH and UKB datasets. The configuration file is openly available^1^ and fully customizable, so it can be adapted to different datasets and expanded to include more variables and conversion rules.

To conclude, we identified processing strategies to maximise the consistency across two large datasets, Whitehall II and UK Biobank, for the study of WMHs. We harmonised non-imaging variables and proposed a processing pipeline to minimise the effect of non-biological sources of difference in the imaging data. The main recommendations emerging from this work are the following:

• Use WMH manual masks generated from the same rater whenever possible and establish guidelines to maximise consistency of the manual masks;
• Perform bias field correction;
• Carefully consider the trade-off between improving segmentation performance with additional modalities (e.g., FA) and using a smaller set of modalities (T1-weighted and FLAIR), which are more reliably present across studies and provide better dataset harmonisation;
• Train BIANCA on data coming from a mix of different scanners/studies when working with more than one dataset.

We showed that with these steps, and appropriate modelling of sample differences through the alignment of demographic, cognitive and physiological variables, we can provide highly consistent WMH measures. These results open up a wide range of applications for the study of WMHs and potentially other neuroimaging markers across extensive databases of clinical data.

## Declaration of Competing Interest

M.J. receives royalties from licensing of FSL to non-academic, commercial entities.

## Appendix Supplementary materials

Image, application 1

## Footnotes

1 https://issues.dpuk.org/eugeneduff/wmh_harmonisation
