# Supplementary material for: Integrating large-scale neuroimaging research datasets: Harmonisation of white matter hyperintensity measurements across Whitehall and UK Biobank datasets
Source: Neuroimage. 2021 Aug 15;237:118189. doi: 10.1016/j.neuroimage.2021.118189 (PMC8285593; doi:10.1016/j.neuroimage.2021.118189)
Supplement: Supplementary file 1 [file mmc1.docx]

**Supplementary material**

**Integrating large-scale neuroimaging research datasets: harmonisation of white matter hyperintensity measurements across Whitehall and UK Biobank datasets**

Valentina Bordin,^a,b^ Ilaria Bertani,^a,b^ Irene Mattioli,^a,c^ Vaanathi Sundaresan,^a^ Paul McCarthy,^a^ Sana Suri,^d,e^ Enikő Zsoldos,^a,d,e^ Nicola Filippini,^d^ Abda Mahmood,^e^ Luca Melazzini,^a,f^ Maria Marcella Laganà,^g^ Giovanna Zamboni,^a,c^ Archana Singh-Manoux,^h,i^ Mika Kivimäki,^i^ Klaus P Ebmeier,^e^ Giuseppe Baselli,^b^ Mark Jenkinson,^a^ Clare E Mackay,^a,d,e,j^ Eugene P Duff,^a,k,*^ Ludovica Griffanti^a,d,#,*^

^a^ Wellcome Centre for Integrative Neuroimaging, Oxford Centre for Functional MRI of the Brain, Nuffield Department of Clinical Neurosciences, University of Oxford, Oxford, UK

^b^ Department of Electronics, Information and Bioengineering, Politecnico di Milano, Milan, Italy

^c^ Department of Biomedical, Metabolic and Neural Sciences, University of Modena and Reggio Emilia, Italy.

^d^ Wellcome Centre for Integrative Neuroimaging, Oxford Centre for Human Brain Activity, Department of Psychiatry, University of Oxford, Oxford, UK

^e^ Department of Psychiatry, Warneford Hospital, University of Oxford, Oxford, UK
^f^ Department of Biomedical Sciences for Health, Università degli Studi di Milano, Milan, Italy

^g^ IRCCS Fondazione Don Carlo Gnocchi ONLUS, Milan, Italy

^h^ INSERM U1153, Epidemiology of Ageing and Neurodegenerative diseases, Université de Paris, Paris, France.
^i^ Department of Epidemiology and Public Health, University College London, London, UK
^j^ Oxford Health NHS Foundation Trust, Oxford, UK
^k^ Department of Paediatrics, University of Oxford, Oxford, UK

* These authors contributed equally to this work

# Corresponding author:

Ludovica Griffanti

Wellcome Centre for Integrative Neuroimaging (WIN)
Department of Psychiatry

Warneford Ln, Headington, Oxford, OX3 7JX

ludovica.griffanti@psych.ox.ac.uk

**SUPPLEMENTARY RESULTS**

***
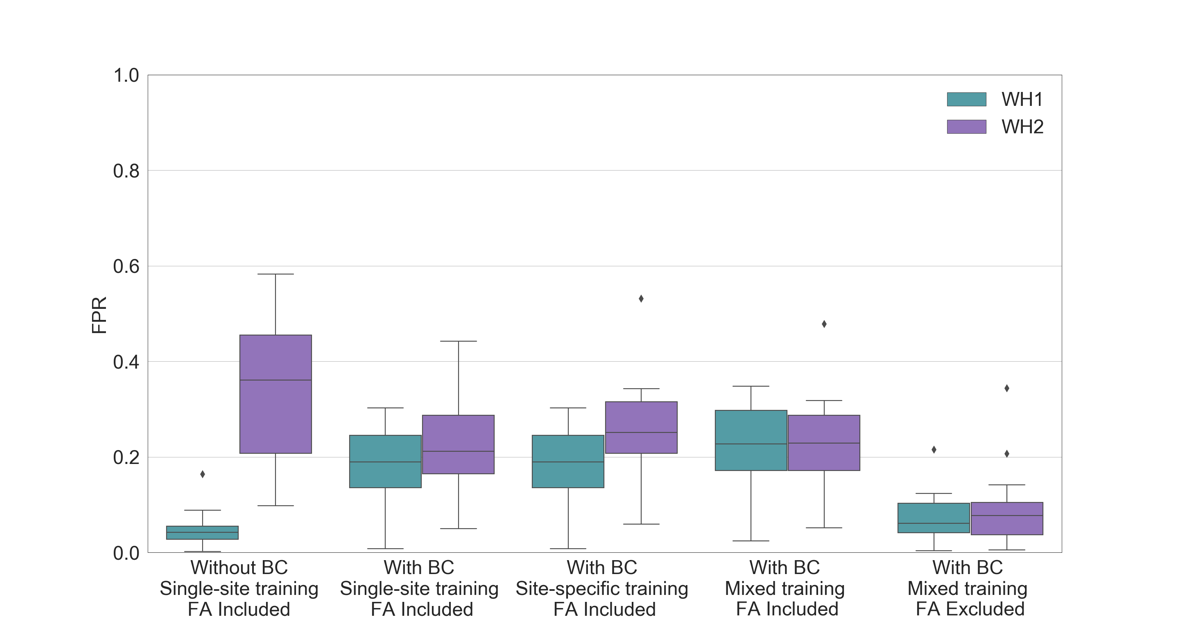
***

***Figure S1.*** *BIANCA performance – scanner upgrade scenario. Box-plot of the voxel-level False Positive Ratio (FPR) between BIANCA output and the corresponding manual masks for the different analysis options tested during our study (specified on the x axis). All the displayed results were evaluated on a sub-sample of manually segmented subjects (12 for WH1 and 12 for WH2) balanced in terms of WMH load and using leave-one-out cross-validation whenever appropriate.*

*
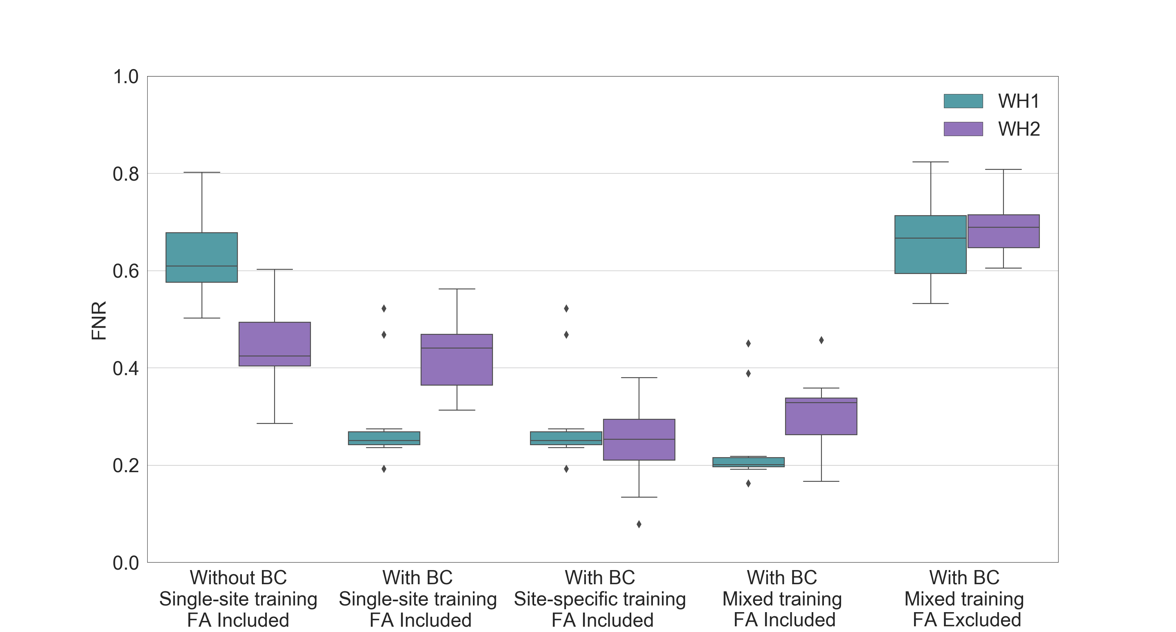
*

***Figure S2.*** *BIANCA performance – scanner upgrade scenario. Box-plot of the voxel-level False Negative Ratio (FNR) between BIANCA output and the corresponding manual masks for the different analysis options tested during our study (specified on the x axis). All the displayed results were evaluated on a sub-sample of manually segmented subjects (12 for WH1 and 12 for WH2) balanced in terms of WMH load and using leave-one-out cross-validation whenever appropriate.*

*
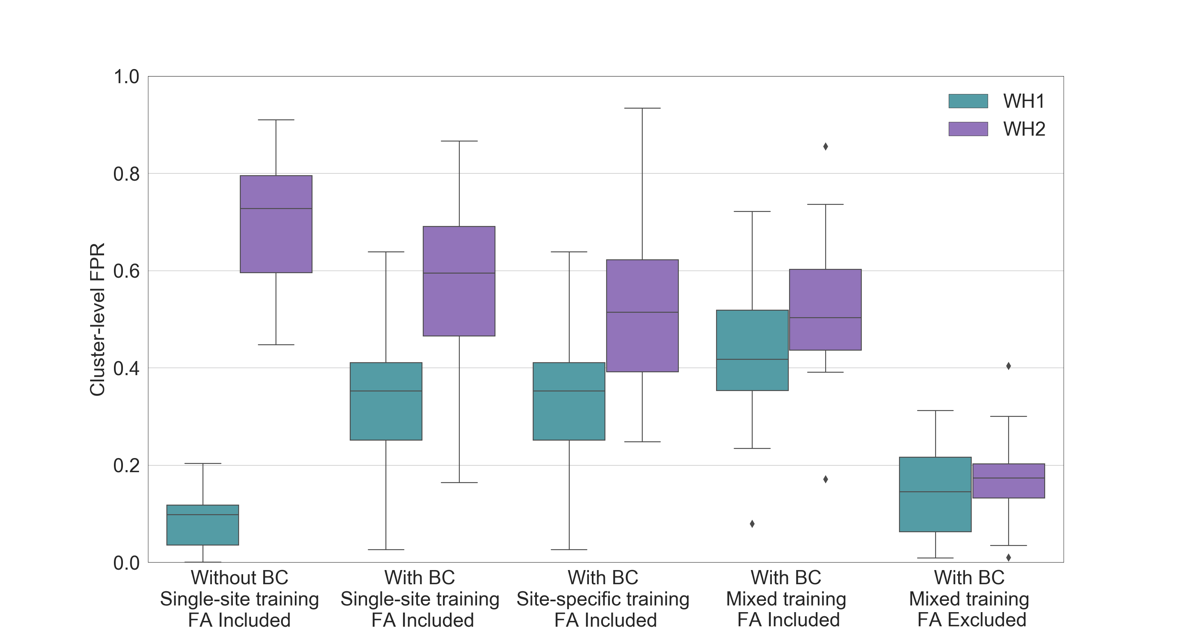
*

***Figure S3.*** *BIANCA performance – scanner upgrade scenario. Box-plot of the cluster-level FPR between BIANCA output and the corresponding manual masks for the different analysis options tested during our study (specified on the x axis). All the displayed results were evaluated on a sub-sample of manually segmented subjects (12 for WH1 and 12 for WH2) balanced in terms of WMH load and using leave-one-out cross-validation whenever appropriate.*

*
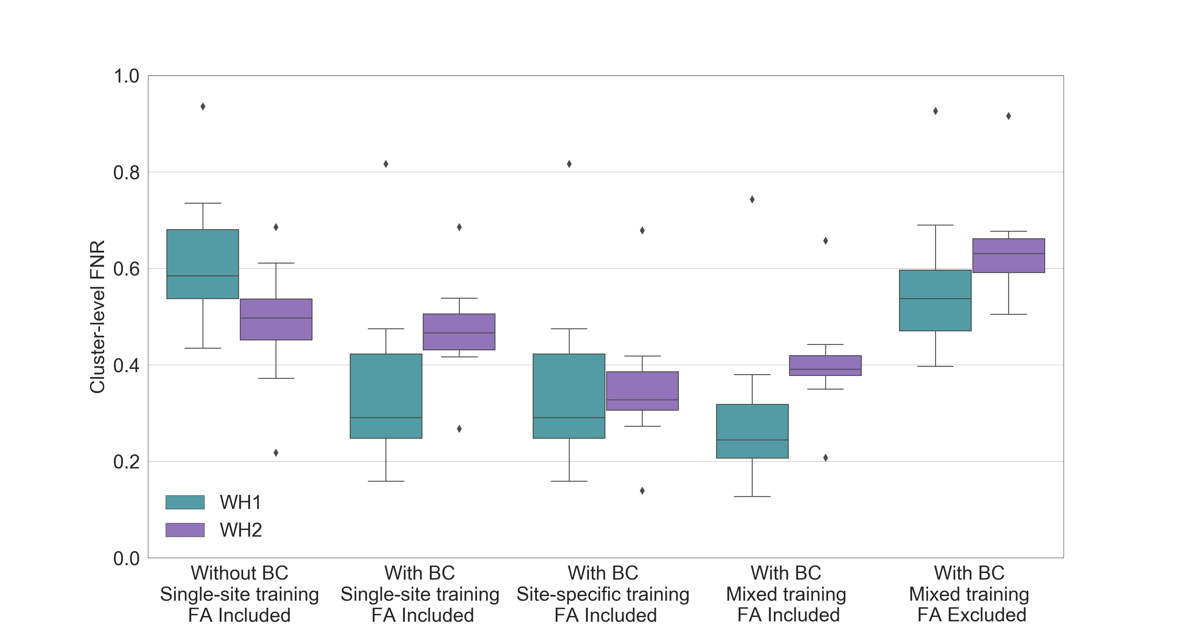
*

***Figure S4.*** *BIANCA performance – scanner upgrade scenario. Box-plot of the cluster-level FNR between BIANCA output and the corresponding manual masks for the different analysis options tested during our study (specified on the x axis). All the displayed results were evaluated on a sub-sample of manually segmented subjects (12 for WH1 and 12 for WH2) balanced in terms of WMH load and using leave-one-out cross-validation whenever appropriate.*

***
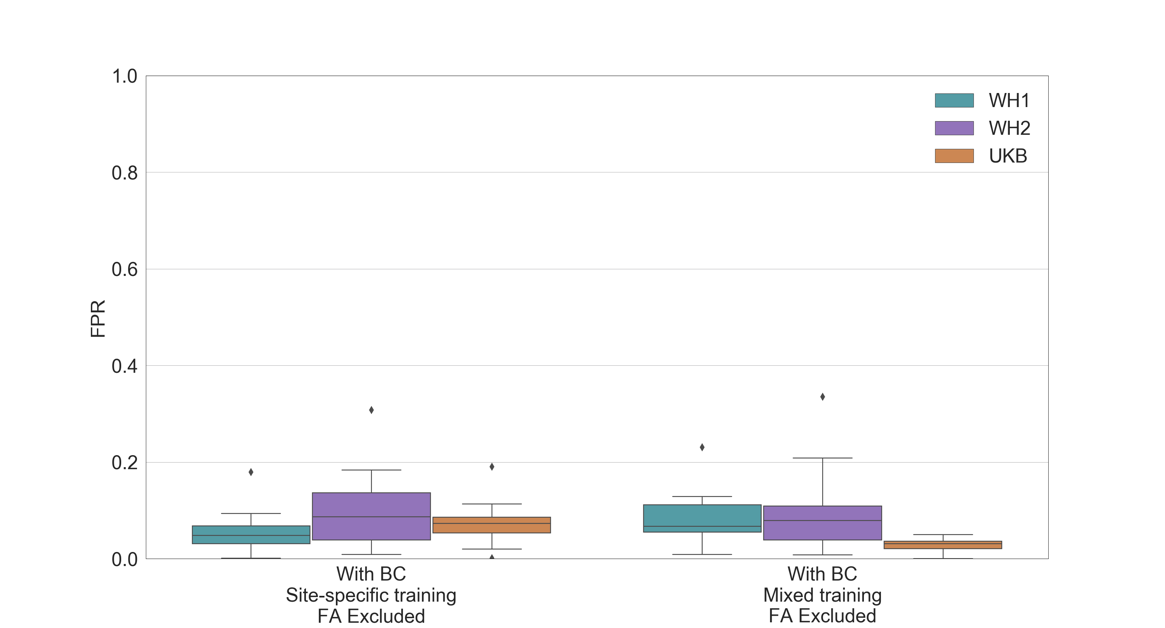
Figure S5.*** *BIANCA performance – retrospective data merging scenario. Box-plot of the voxel-level False Positive Ratio (FPR) between BIANCA output and the corresponding manual mask for the different analysis options tested during our study (specified on the x axis) All the displayed results were evaluated on a sub-sample of manually segmented subjects (12 for WH1, 12 for WH2 and 12 for UKB) balanced in terms of WMH load and using leave-one-out cross-validation whenever appropriate.*

*
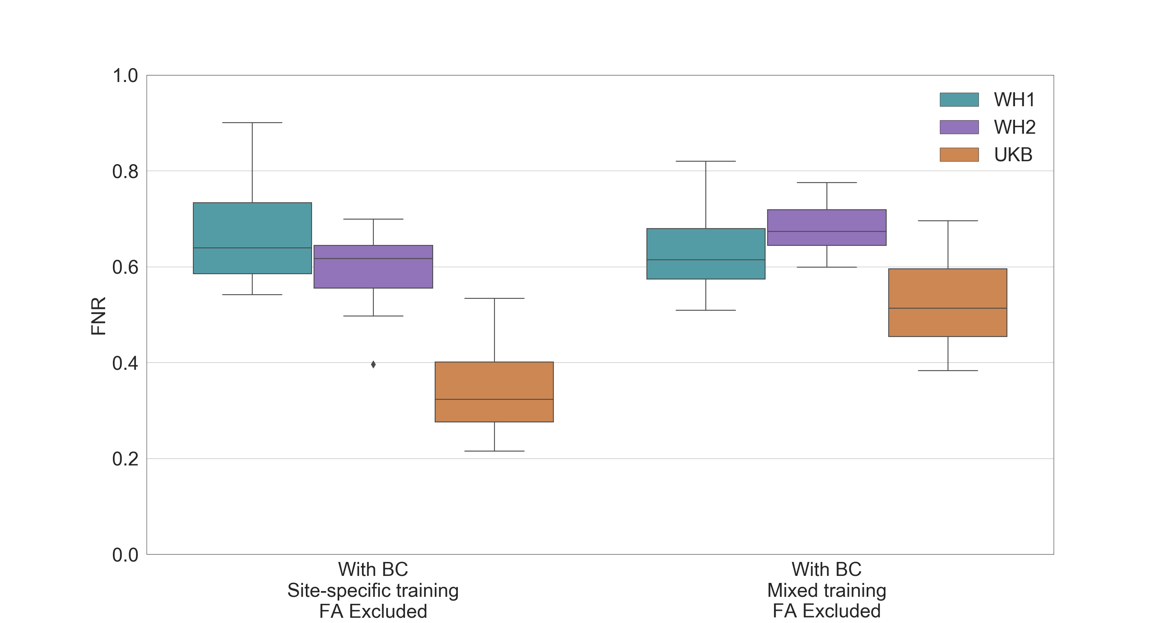
*

***Figure S6.*** *BIANCA performance – retrospective data merging scenario. Box-plot of the voxel-level False Negative Ratio (FNR) between BIANCA output and the corresponding manual mask for the different analysis options tested during our study (specified on the x axis) All the displayed results were evaluated on a sub-sample of manually segmented subjects (12 for WH1, 12 for WH2 and 12 for UKB) balanced in terms of WMH load and using leave-one-out cross-validation whenever appropriate.*

*
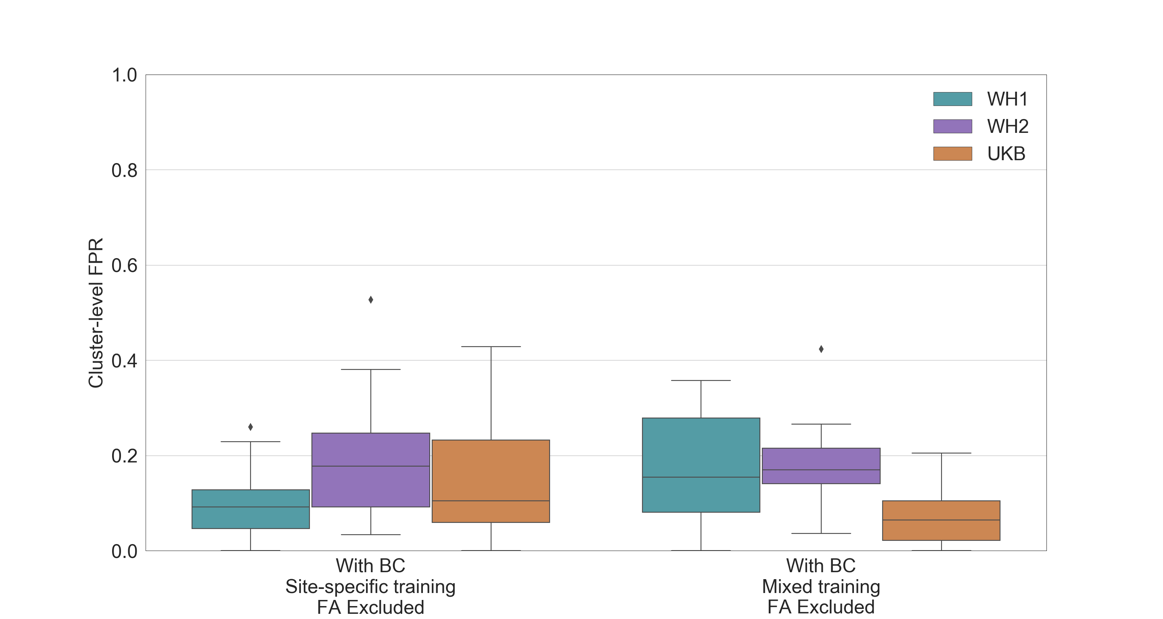
****Figure S7.*** *BIANCA performance – retrospective data merging scenario. Box-plot of the cluster-level FPR between BIANCA output and the corresponding manual mask for the different analysis options tested during our study (specified on the x axis) All the displayed results were evaluated on a sub-sample of manually segmented subjects (12 for WH1, 12 for WH2 and 12 for UKB) balanced in terms of WMH load and using leave-one-out cross-validation whenever appropriate.*

*
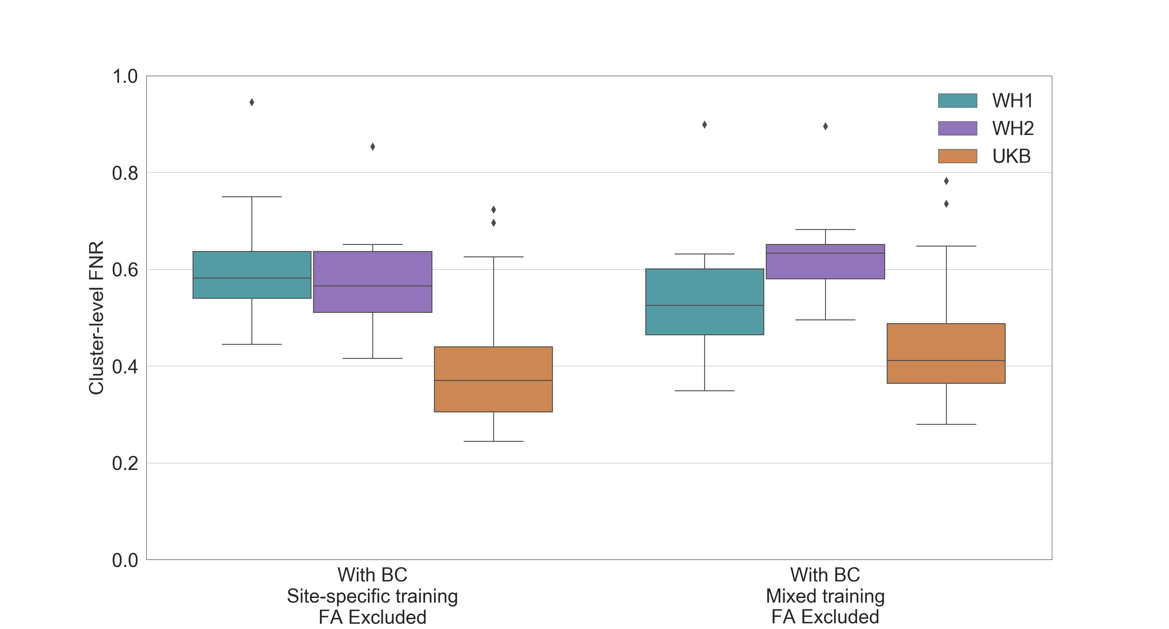
*

***Figure S8.*** *BIANCA performance – retrospective data merging scenario. Box-plot of the cluster-level FNR between BIANCA output and the corresponding manual mask for the different analysis options tested during our study (specified on the x axis). All the displayed results were evaluated on a sub-sample of manually segmented subjects (12 for WH1, 12 for WH2 and 12 for UKB) balanced in terms of WMH load and using leave-one-out cross-validation whenever appropriate.*

**Table S1.** Details of ‘traveling heads’ participants.

| **ID** | **Sex** | **Age at scan on scanner SC1** | **Days between scans** | **Time of the day scanner SC1** | **Time of the day scanner SC2** |
| --- | --- | --- | --- | --- | --- |
| P01 | Male | 39 | 140 | 7:53 | 14:51 |
| P03 | Female | 26 | 151 | 10:25 | 15:42 |
| P04 | Female | 31 | 153 | 11:14 | 14:48 |
| P05 | Male | 28 | 153 | 12:12 | 16:14 |
| P06 | Female | 31 | 152 | 13:02 | 8:59 |

*SC1: 3T Siemens Verio; SC2: 3T Siemens Prisma*

**Table S2.** Effect of rater – Summary of Dice Similarity Index measures calculated for the agreement between manual masks annotated by the raters and BIANCA outputs generated with masks from those raters. Statistical tests performed on data to assess the impact of between- and within-rater variability on the segmentation performance.

|  |  | **R1 vs R2a (between-rater variability)** | | **R1 vs R2b (between-rater variability)** | | **R2a vs R2b (within-rater variability)** | | |
| --- | --- | --- | --- | --- | --- | --- | --- | --- |
|  |  | Manual (M1 vs M2a) | BIANCA (B1 vs B2a) | Manual (M1 vs M2b) | BIANCA (B1 vs B2b) | Manual (M2a vs M2b) | BIANCA (B2a vs B2b) |  |
| **Overlap measures** | **Mean ± std** | 0.54 ± 0.18 | 0.43 ± 0.13 | 0.60 ± 0.13 | 0.69 ± 0.10 | 0.54 ± 0.22 | 0.63 ± 0.11 |  |
| **Within-subject analysis:** paired t-test | **Manual vs BIANCA** | < 0.001 *** | | < 0.001 *** | | < 0.001 *** | |  |

*Results relative to the statistical tests are all reported in terms of p-values: * (< 0.05), ** (< 0.01), *** (< 0.001). Legend: R1 = rater 1, R2a = Rater 2, first rating, R2b = rater 2, second rating (1 year apart from the first rating, blind to first rating), M=manual, B=BIANCA.*

**Table S3.** Elastic Net Regression performance – scanner upgrade and retrospective data merging scenario – Summary of the results in terms of variance explained by the model and by a subset of the features which constituted it. All variables are here reported for the different analysis options tested in our study.

|  |  | **Scanner upgrade scenario** | | | | | **Retrospective data merging scenario** | |
| --- | --- | --- | --- | --- | --- | --- | --- | --- |
|  |  | **Analysis option A** | **Analysis option B** | **Analysis option C** | **Analysis option D** | **Analysis option E** | **Analysis option A** | **Analysis option B** |
| **Variance explained by the model** | | 0.243 | 0.161 | 0.207 | 0.173 | 0.125 | 0.190 | 0.098 |
| **Variance explained by the features** | **Age** | 0.060 | 0.044 | 0.049 | 0.054 | 0.034 | 0.052 | 0.070 |
|  | **Sex** | 0.014 | 0.014 | 0.004 | 0.014 | 0.006 | 0.003 | 0.006 |
|  | **Width** | ------- | ------- | ------- | ------- | 0.000 | ------- | ------- |
|  | **Height** | 0.012 | 0.006 | ------- | 0.010 | 0.006 | 0.008 | 0.008 |
|  | **Body Mass Index** | ------- | 0.003 | 0.005 | 0.002 | 0.005 | ------- | 0.003 |
|  | **Systolic Blood Pressure** | 0.004 | 0.013 | 0.007 | 0.013 | 0.006 | ------- | ------- |
|  | **Diastolic Blood Pressure** | 0.010 | 0.005 | 0.014 | 0.010 | 0.000 | 0.011 | 0.017 |
|  | **Pulse Rate** | ------- | ------- | ------- | ------- | ------- | ------- | ------- |
|  | **Moderate Physical Activity** | ------- | ------- | ------- | ------- | ------- | ------- | 0.004 |
|  | **Vigorous Physical Activity** | ------- | 0.001 | 0.002 | ------- | ------- | ------- | ------- |
|  | **CHAM Walk** | 0.002 | ------- | ------- | ------- | ------- | 0.007 | 0.005 |
|  | **TV** | 0.005 | ------- | ------- | ------- | ------- | ------- | ------- |
|  | **Total Walking Activity** | ------- | ------- | ------- | 0.004 | ------- | ------- | ------- |
|  | **Sleep Duration** | 0.006 | ------- | 0.002 | ------- | 0.000 | ------- | ------- |
|  | **Health Classes** | ------- | ------- | ------- | ------- | ------- | ------- | ------- |
|  | **Smoker Status** | ------- | ------- | ------- | ------- | ------- | ------- | ------- |
|  | **Smoking Units** | ------- | ------- | ------- | ------- | ------- | ------- | ------- |
|  | **Alcohol Status** | ------- | ------- | ------- | ------- | ------- | 0.007 | 0.003 |
|  | **Alcohol Units** | ------- | ------- | 0.004 | 0.004 | ------- | ------- | ------- |
|  | **Total Medications** | ------- | 0.005 | ------- | ------- | ------- | 0.009 | 0.008 |
|  | **Cardiovascular Disease - Medications** | 0.013 | 0.008 | 0.011 | 0.019 | 0.009 | ------- | ------- |
|  | **Depression - Medications** | ------- | ------- | 0.008 | ------- | ------- | ------- | ------- |
|  | **Blood Pressure - Medications** | 0.011 | 0.013 | 0.009 | 0.009 | 0.007 | 0.006 | 0.014 |
|  | **Diabetes** | 0.004 | 0.010 | ------- | 0.008 | 0.008 | 0.004 | 0.005 |
|  | **Cardiovascular Disease** | ------- | ------- | ------- | ------- | 0.001 | 0.015 | 0.014 |
|  | **Depression** | ------- | ------- | ------- | 0.004 | ------- | ------- | ------- |
|  | **Education** | ------- | ------- | ------- | ------- | ------- | ------- | ------- |
|  | **Hand – Class** | ------- | ------- | 0.003 | ------- | ------- | 0.007 | 0.006 |
|  | **Trail Making Test - A** | ------- | ------- | ------- | ------- | ------- | 0.006 | 0.012 |
|  | **Trail Making Test - B** | 0.003 | ------- | ------- | ------- | 0.007 | 0.008 | 0.020 |
|  | **Digit Span Backward** | ------- | 0.001 | ------- | ------- | ------- | 0.009 | ------- |
|  | **Digit CODing** | 0.009 | 0.016 | 0.013 | 0.017 | 0.010 | ------- | ------- |
|  | **Reaction Time** | 0.022 | 0.010 | 0.006 | 0.012 | 0.011 | ------- | ------- |
|  | **Scanner** | 0.047 | 0.013 | 0.067 | 0.009 | ------- | 0.115 | ------- |

*Options tested in our study are: (I) for the scanner upgrade scenario: (A) without BC, single-site training, FA included; (B) with BC, single-site training, FA included; (C) with BC, site-specific training, FA included; (D) with BC, mixed training, FA included; (E) with BC, mixed training, FA excluded; (II) for the retrospective data merging scenario: (A) with BC, site-specific training, FA excluded; (B) with BC, mixed training, FA excluded. The amount of WMH variance explained by the model is calculated using the R-squared coefficient and reported in the first row. The amount of WMH variance explained by the features is reported in the rest of the table for all variables.*

**SUPPLEMENTARY ANALYSES**

**Mixed training set composition**

To better understand the results obtained in our main analysis, we investigated how the information from the mixed training dataset was used by BIANCA. In fact, even if the training points come from different datasets, only the (k=40) neighbours are used in the classification of each point. Therefore, theoretically it is not impossible that training points belonging to the different datasets would be so far apart in the features space that effectively the kNN classifier would use only nearest neighbours from the specific dataset and effectively not use a mixed set of training points.

While BIANCA doesn’t natively store the information about the sites of the points used for the prediction, we performed the following experiment.

We modified BIANCA code to store the information about site for each point used to create the mixed training dataset and, for a test subject, to save the features of each point, together with the site information.

We then trained a kNN (k=40, as used in BIANCA) to learn the site label from the features of the training points and tested it on a subset of test subjects from each scanner.

For each point of the test images we calculated the proportion of neighbours from the three different datasets used by the kNN classifier (ahead of thresholding). Our hypothesis was that if a proportion of the neighbours came from different datasets than the one of the testing subject, that would suggest a certain degree of features mixing (effectively harmonisation happening).

We tested a subset of subjects for each scanner, including the youngest subject, oldest subject, subject with the highest lesion load, subject with the lowest lesion load, and 2 subjects from the age range common to the three datasets (see also next section about this).

Figure S9 shows a boxplot of the proportion of neighbours from different datasets for each voxel of the tested subjects, while Table S4 reports the mean and standard deviation across voxels. While the highest proportion of neighbours tended to come from the same site as the test subject, in all cases there were neighbours from other sites. The age and lesion load doesn’t seem to have an effect on the neighbours’ proportion.

*****Figure S9****. Mixed training set composition – Boxplot of the proportion (range 0-1) of neighbours belonging to different datasets (specified on the x axis: WH1 = cyan; WH2 = purple; UKB = orange) calculated for each voxel of the tested subjects (datasets of belonging specified on the y axis). For each dataset, we tested: (i) the youngest subject, (ii) the oldest subject, (iii) the subject with the highest lesion load, (iv) the subject with the lowest lesion load, and (v; vi) 2 subjects from the age range common to the three datasets.*

| **Subject dataset** | **Neighbourhood dataset** | **Minimum age subject** | **Maximum age subject** | **Minimum WMH load subject** | **Maximum WMH load subject** | **Random age-matched subject** | |
| --- | --- | --- | --- | --- | --- | --- | --- |
| **WH1** | **WH1** | 0.57 ± 0.20 | 0.48 ± 0.19 | 0.61 ± 0.19 | 0.44 ± 0.19 | 0.60 ± 0.19 | 0.53 ± 0.21 |
|  | **WH2** | 0.31 ± 0.21 | 0.38 ± 0.20 | 0.27 ± 0.20 | 0.37 ± 0.20 | 0.28 ± 0.19 | 0.34 ± 0.21 |
|  | **UKB** | 0.12 ± 0.13 | 0.14 ± 0.13 | 0.12 ± 0.13 | 0.19 ± 0.18 | 0.12 ± 0.13 | 0.12 ± 0.14 |
| **WH2** | **WH1** | 0.29 ± 0.20 | 0.26 ± 0.17 | 0.27 ± 0.18 | 0.35 ± 0.18 | 0.28 ± 0.18 | 0.39 ± 0.20 |
|  | **WH2** | 0.61 ± 0.25 | 0.65 ± 0.21 | 0.65 ± 0.22 | 0.49 ± 0.22 | 0.62 ± 0.23 | 0.50 ± 0.24 |
|  | **UKB** | 0.10 ± 0.13 | 0.09 ± 0.11 | 0.08 ± 0.12 | 0.17 ± 0.16 | 0.10 ± 0.13 | 0.11 ± 0.13 |
| **UKB** | **WH1** | 0.35 ± 0.18 | 0.32 ± 0.18 | 0.36 ± 0.20 | 0.33 ± 0.19 | 0.31 ± 0.18 | 0.35 ± 0.19 |
|  | **WH2** | 0.28 ± 0.21 | 0.27 ± 0.18 | 0.26 ± 0.19 | 0.26 ± 0.20 | 0.26 ± 0.19 | 0.29 ± 0.21 |
|  | **UKB** | 0.37 ± 0.21 | 0.42 ± 0.22 | 0.38 ± 0.22 | 0.40 ± 0.22 | 0.42 ± 0.22 | 0.37 ± 0.21 |

**Table S4** – Analysis of the mixed training set composition – Summary of the proportion of neighbours belonging to different datasets (WH1, WH2 and UKB) calculated across all voxels of the tested subjects.

*For each dataset, we tested: (i) the youngest subject, (ii) the oldest subject, (iii) the subject with the highest lesion load, (iv) the subject with the lowest lesion load, and (v; vi) 2 subjects from the age range common to the three datasets. For each subject we reported mean ± std values.*

**Age matching**

To verify that results from the retrospective data merging scenario were not driven by the age difference across the three datasets, we conducted the following analyses.

We explored if there was a relationship between BIANCA performance (Dice) and age for the subjects for which we had manual masks (Figure S10).

Both before and after harmonisation we did not observe a significant correlation between age and BIANCA performance. The Spearman’s correlation coefficient was indeed equal to -0.098 (p-value = 0.570) in the first case and equal to -0.248 (p-value = 0.145) in the second.

Moreover, we repeated the analyses presented in Figure 7, 8 and Table 5, 6 on an equal subset (N=170) of subjects, age-matched across the 3 datasets (age-range=65-77). The results, presented below, highlighted a similar pattern with respect to the original analyses, suggesting that no significant bias was introduced in our initial findings by the domain mismatch in the UKB and WH datasets. The one-way ANCOVA test performed on the correlation between WMH volumes and age showed no significant differences in the slope of the regression lines for both the non-harmonised and harmonised case. Results also highlighted a significant decrease in the volume bias that was characterising the non-harmonised data, after the adoption of a mixed training strategy (Figure S11, Table S5).

Additionally, the implemented Elastic Net model showed that the scanner/site variable was the feature explaining the greatest amount of variance for the site-specific option, while it was no longer present amongst predictive features after harmonisation (Figure S12, Table S6).

Even though we cannot exclude the possibility of some volume over/underestimation especially in the younger subjects, the observed volume differences seem not to be driven by subjects’ age or by differences in age range across datasets. Future evaluations on additional samples/datasets of younger subject and with manual masks available will be important to further investigate this aspect.

***Figure S10****. Association between Dice Similarity Index (DI) and age – retrospective data merging scenario. Scatter plot of the relationship between DI (y axis) and age (x axis), for WH1 (cyan), WH2 (purple) and UKB (orange) data. Regression lines with 95% confidence interval are also displayed. Each plot refers to one of the investigated analysis options: (A) with BC, site-specific training, FA excluded; (B) with BC, mixed training, FA excluded. Evaluation was conducted on a sub-sample of manually segmented subjects (12 for WH1, 12 for WH2 and 12 for UKB) balanced in terms of WMH load.*

***Figure S11.*** *Association between WMHs and age – retrospective data merging scenario. Scatter plot of the relationship between WMH volumes (expressed as % of total brain volume, y axis) and age (x axis), for WH1 (cyan), WH2 (purple) and UKB (orange) data. Regression lines with 95% confidence interval are also displayed. Each plot refers to one of the investigated analysis options: (A) with BC, site-specific training, FA excluded; (B) with BC, mixed training, FA excluded. Evaluation was conducted on a sub-sample of age-matched participants across the three datasets (WH1 = 170, WH2 = 170, UKB = 170).*

***Figure S12.*** *Multivariate model – retrospective data merging scenario. Percentage of variance (reported on the y axis) explained by non-imaging variables (reported on the x axis) in the linear multivariate model that was implemented (Elastic Net). Evaluation was conducted on a sub-sample of age-matched participants across the three datasets (WH1 = 170, WH2 = 170, UKB = 170). Each plot refers to one of the investigated analysis options: (A) with BC, site-specific training, FA excluded; (B) with BC, mixed training, FA excluded. Variable scanner/site (SC) highlighted in red.*

|  | **Retrospective data merging scenario** | |
| --- | --- | --- |
|  | **Analysis option A** | **Analysis option B** |
| **One-way ANCOVA Slope** | F(1, 506) = 1.006, p = 0.316 | F(1, 506) = 1.779, p = 0.183 |
| **One-way ANCOVA Intercept** | F(1, 506) = 135.228, p < 0.001 *** | F(1, 506) = 0.365, p = 0.546 |

**Table S5.** Analysis of the relationship between WMH volumes and age – scanner upgrade and retrospective data merging scenario – Summary of the one-way ANCOVA test.

*Options tested in our study are, for the retrospective data merging scenario: (A) with BC, site-specific training, FA excluded; (B) with BC, mixed training, FA excluded. The one-way ANCOVA test evaluated across-scanner differences (between WH1/WH2/UKB) characterising regression slope (interaction between age and scanner) and intercept at mean age (main effect of scanner) in the linear model relating WMH% to age. Results are reported in terms of F(df)- and p-values: * (< 0.05), ** (< 0.01), *** (< 0.001).*

**Table S6.** Elastic Net Regression performance – retrospective data merging scenario – Summary of the results in terms of variance explained by the model and by age and scanner, the features considered most relevant in our study.

|  | | **Retrospective data merging scenario** | |
| --- | --- | --- | --- |
|  | | **Analysis option A** | **Analysis option B** |
| **Variance explained by the model** | | 0.123 | 0.056 |
| **Variance explained by the features** | **Age** | 0.041 | 0.020 |
|  | **Scanner** | 0.117 | 0.000 |

*Options tested are, for the retrospective data merging scenario: (A) with BC, site-specific training, FA excluded; (B) with BC, mixed training, FA excluded. The amount of WMH variance explained by the model is calculated using the R-squared coefficient and reported in the first row. The amount of WMH variance explained by the features is reported in the rest of the table for the most relevant variables (age and scanner).*
